# Influence of data acquisition modes and data analysis approaches on non-targeted analysis of phthalate metabolites in human urine

**DOI:** 10.1007/s00216-022-04407-7

**Published:** 2022-11-08

**Authors:** Yong-Lai Feng, Anca Baesu

**Affiliations:** Exposure and Biomonitoring Division, Environmental Health Science and Research Bureau, Environmental and Radiation Health Sciences Directorate, Healthy Environments and Consumer Safety Branch, Health Canada, AL: 2203 B, 251 Sir Frederick Banting Driveway, Ottawa, ON K1A 0K9 Canada

**Keywords:** Non-targeted analysis (NTA), Data acquisition mode, Phthalate metabolites, Data analysis approach, Human urine

## Abstract

Humans are often exposed to phthalates and their alternatives, on account of their widespread use in PVC as plasticizers, which are associated with harmful human effects. While targeted biomonitoring provides quantitative information for exposure assessment, only a small portion of phthalate metabolites has been targeted. This results in a knowledge gap in human exposure to other unknown phthalate compounds and their metabolites. Although the non-targeted analysis (NTA) approach is capable of screening a broad spectrum of chemicals, there is a lack of harmonized workflow in NTA to generate reproducible data within and between different laboratories. The objective of this study was to compare two different NTA data acquisition modes, the data-dependent (DDA) and independent (DIA) acquisition (DDA), as well as two data analysis approaches, based on diagnostic ions and Compound Discoverer software for the prioritization of candidate precursors and identification of unknown compounds in human urine. Liquid chromatography coupled to high-resolution mass spectrometry was used for sample analysis. The combination of three-diagnostic-ion extraction and DDA data acquisition was able to improve data filtering and data analysis for prioritizing phthalate metabolites. With DIA, 25 molecular features were identified in human urine, while 32 molecular features were identified in the same urine samples using DDA data. The number of molecular features identified with level 1 confidence was 11 and 9 using DIA and DDA data, respectively. The study demonstrated that besides sample preparation, the impact of data acquisition must be taken into account when developing a NTA method and a consistent protocol for evaluating such an impact is necessary.

**Graphical Abstract:**

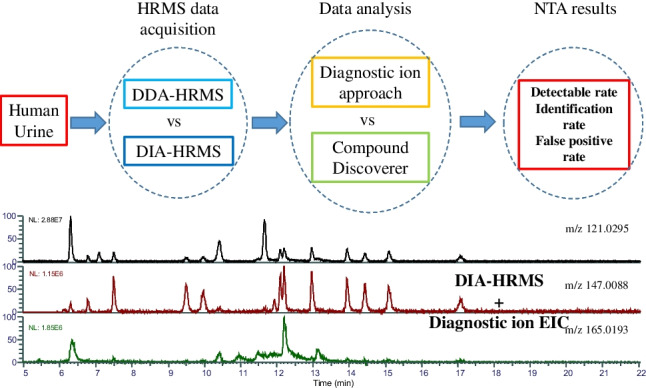

**Supplementary Information:**

The online version contains supplementary material available at 10.1007/s00216-022-04407-7.

## Introduction

Phthalates are a group of chemicals commonly used as plasticizers in PVC materials [1, 2] and as solvents in many consumer products and cosmetics [3]. Human exposure to this group of chemicals has been associated with health issues, including altered hormone concentrations, reproductive and developmental effects, some chronic diseases, and development of some cancers [4–8]. As a result, some phthalates have been banned or restricted in many countries, including Europe [9], and Canada [10], and replaced by new phthalates or alternative plasticizer compounds, most of which are unknown. Quantitative biomonitoring is a common way to provide information on human exposure to phthalates [8, 11]. Target sample preparation methods for monophthalates include dispersive solid phase extraction [12], solvent extraction [13], and solid phase extraction [14]. Among some quantitative biomonitoring programs, the Canadian Health Measurement Survey (CHMS) project (CHMS, 2007–current) aims to characterize Canadians’ chemical exposure, to provide evidence of possible environmental health effects and to support public policy making [15]. However, the targeted quantitative methods included in the CHMS project, only cover a small portion of phthalates metabolites, since the number of analytical standards used in method development is limited. Hence, there is a need to extend the range of detected markers of exposure such as metabolites, and to generate a broader picture of human exposure to phthalates and unknown alternatives for exposure assessment.

Recently, non-targeted analysis (NTA) using high-resolution mass spectrometry (HRMS) has been attracting interest in exposure assessment of emerging contaminants and health- and disease-related issues [16]. NTA takes advantage of the information obtained using HRMS, e.g., accurate mass, to general formulas for compounds of interest, as well as elucidate structures using fragmentation information [17]. Further data processing may include statistical analysis that allows prioritizing specific molecular features for structural elucidation [18]. Due to this ability to detect more compounds present in a sample, NTA can help identify known compounds, sometimes referred to as suspect screening, as well as compounds for which no information is available, referred to as “unknown unknowns” such as degradation products or metabolites [19]. For example, a non-targeted screening method was developed to characterize halogenated compounds in human milk for assessment of human chemical exposure [20] with full scan data in both negative and positive ionization modes acquired for data analysis with defined peak-picking parameters. Monoisotopic ion masses matched with a known compound list, after blank subtraction, were considered detected in milk. However, there was no characterization information for those masses. In another study, a non-targeted method based on comprehensive two-dimensional gas chromatography coupled to time-of-flight mass spectrometry was used to screen organic contaminants in human breast milk, and 172 presumably anthropogenic halogenated, non-halogenated cyclic, and aromatic compounds were tentatively identified with the NTA-based method using the NIST mass spectral library [21]. Besides metabolite screening and identification, the NTA approach has been used to screen and identify DNA adducts as markers of human exposure to genotoxic contaminants [22–24]. However, NTA approaches still remain challenging. Often, compound identification is based on available databases. Although these databases are regularly updated with mass, MS/MS spectra, etc., they often lack information on “unknown unknown” compounds, and are therefore insufficient for chemical identification of truly unknown compounds. In addition, LC-HRMS-based MS/MS spectra in existing libraries, e.g., different ionization, and collision energies, have not been standardized, which further leads to false positives in NTA identification. Meanwhile, the lack of harmonization and reporting of analytical workflows and method performance assessment for suspect and non-target screening compromises the reliability and credibility of NTA results. This makes it difficult to achieve repeatable and comparable results between laboratories, which hinders the interpretation of the results, thereby affecting risk assessment and policy making.

The NTA community has currently devoted many efforts to standardize these workflows and provide guidelines for reporting NTA methods and results, which aims to be able to provide comparable qualitative and quantitative information for exposure and risk assessment [25–28]. However, these new methodological approaches still face a number of limitations and challenges [29]. Other than the sample preparation step, where a compromise must be reached between sufficient selectivity to remove matrix interfering compounds while retaining compounds of interest, data acquisition methods and data analysis approaches applied in NTA are two other key steps that have not been yet harmonized to achieve reproducible results. Full scan, data-dependent acquisition (DDA) and data-independent acquisition (DIA) are acquisition modes applied in NTA. In full scan, information is obtained for all ions in a sample, and although the accurate mass and isotopic abundance are sufficient to generate formulas for compounds, there is no fragmentation within the mass spectrometer that would enable to increase the confidence level of identification using MS/MS spectra [17]. With DDA, precursor ions are selected for fragmentation, based on specific criteria such as intensity or an inclusion list. On the other hand, DIA can acquire MS/MS spectra for all ions, with no predefined criteria that would trigger ion fragmentation [30]. DIA modes can include SIM (selected ion monitoring), where selected ions are fragmented within target mass windows [31], or all-ion fragmentation where all ions are selected for fragmentation [30]. Previous studies have reported the performance of different data acquisition modes for NTA, like suspect screening of known analytes such as pesticides and veterinary drugs in foods [30, 32] or urine metabolomics [33]. Advantages and disadvantages are associated with each acquisition mode. For example, a full scan coupled to SIM can increase sensitivity for some target analytes [31]. When compounds of interest are present at very low concentrations, DDA may not be pertinent as the intensities are not sufficient to trigger the fragmentation required for identification. Data processing also varies between studies, and there is often limited information on how data processing parameters were chosen. To date, few studies have been conducted on the non-targeted identification of phthalate metabolites and the comparison of different acquisition modes and data analysis approaches for NTA identification and prioritization of this group of chemicals. Therefore, in this study, we aimed to assess the impact of two data acquisition modes, DDA vs. DIA, and two different non-targeted data analysis approaches (Compound Discoverer vs. diagnostic ions), on the identification and prioritization of phthalate metabolites in urine samples.

## Materials and methods

### Chemicals

Methanol and acetonitrile (ACN) were obtained from VWR Canada (Mississauga, Ontario, Canada). Ethyl acetate, β-glucuronidase (type VII-A, lyophilized powder, 5292 U/mg) from *Escherichia coli*, acetic acid (> 99.7%), 4-methylumbelliferyl β-D-glucuronide hydrate (4-MUGH, > 98%), and ammonium acetate (> 99.0%) were purchased from Sigma-Aldrich (St Louis, MO). Milli-Q deionized water (DIW) (resistivity 18.2 M) made in-house with a Milli-Q Reference A + water purification system from Millipore (Bedford, MA, USA).

### Preparation of solutions

Each stock solution of the 24 native standards and 14 isotope labelled standards, in methyl tert-butyl ether (MTBE) (100 µg/mL, Tables S1 and S2), was obtained from Cambridge Isotope Laboratories. The working mixture solution (2 µg/mL) was prepared by combining each isotope standard solution (100 µg/mL) followed by evaporation of MTBE with a gentle flow of nitrogen and reconstitution in 20% acetonitrile in water. The stock and working solutions were kept at − 20 °C.

### Sample preparation

The study was approved by the Research Ethics Board of Health Canada (REB 2017–0023). Two pooled urine samples, collected during a man fertility study [8], were prepared by mixing equal volumes of urine in each group. The two groups of urine samples, collected from fertile men (control-106 samples) and infertile men (45), were collected in Montreal, Canada. Samples were stored at − 80 °C. One milliliter of pooled urine that was naturally thawed at room temperature was transferred to a 15-mL polypropylene tube. To each sample, 50 µL of 2 ppm labelled standard working mixture solution (14 labelled monophthalate metabolites) was added, followed by 250 µL of 1 M ammonium acetate buffer (pH = 6.5), and 100 µL of 2800 U/mL of β-glucuronidase solution (in 1 M ammonium acetate buffer). The mixture was incubated at 37 °C for 120 min in an Eppendorf Thermomixer 5430R (Eppendorf, Hamburg, Germany) to hydrolyze the conjugated metabolites; the temperature was increased to 60 °C for another 15 min to deactivate the enzyme. After incubation, samples were cooled to room temperature and 250 µL of acetonitrile was added. The mixture was extracted three times using 500 µL of ethyl acetate. The ethyl acetate extracts were combined and evaporated to dryness with a gentle flow of nitrogen gas. The residues were reconstituted with 150 µL of 20% acetonitrile/water solution (v/v) for injection using the Thermo Fisher Vanquish UHPLC-Q-Exactive MS system. For lab blank control, 1 ml of DIW water was used instead of urine. All other reagents were the same as urine samples and the same sample preparation procedure was followed. Three replicates were prepared for each pooled urine sample, i.e., fertile and infertile men, and lab blanks.

### UHPLC-MS conditions

All data acquisition was performed using a Thermo Fisher Q-Exactive Orbitrap mass spectrometer with an electrospray ionization source in negative mode (Thermo Fisher Sientific Inc., Waltham, MA, USA), coupled with a Thermo Fisher Vanquish Horizon UHPLC system (Thermo Fisher Scientific Inc. Mississauga, Canada). Mass calibration was performed weekly, per the manufacturer’s instructions, using Thermo Fisher Pierce ESI Negative Ion Calibration Solution, using the following reference ions: 68.99576, 96.96010, 265.14790, 514.28440, 1180.00360, 1279.99720, and 1379.99080. A Kinetex biphenyl column (2.1 × 150 mm, 2.6 μm) (Phenomenex, Torrance, CA, USA) with a SecurityGuard ULTRA cartridge (2.1 × 2 mm) (Phenomenex, Torrance CA, USA) was used. Mobile phases were A (0.1% formic acid in DIW) and B (0.1% formic acid in methanol). The separation was performed with a solvent gradient as follows: start at 15% B and hold for 1 min; increase to 58% B over 2.5 min, and hold for 5.5 min; increase to 75% B over 1 min and hold for 7 min; increase to 85% B over 3 min and hold for 16 min; decrease back to 15% B over 1 min. The equilibrium time was 13 min before the next injection. The flow rate of the mobile phase was 0.28 mL/min, and the injection volume was 10 µL. The column temperature was set at 40 °C. Different data acquisition modes were used for different sections of this work, which are described below. Samples were run in negative mode ionization only, as the focus of the study was on phthalate metabolites, which ionize primarily in negative mode, with a sequence of two water blanks, QC blanks, QC spiked samples, two water blanks, and real samples.

### Data acquisition for non-targeted analysis of urine samples

For data-independent acquisition (DIA) mode, the samples were analyzed in negative ionization mode using full-scan SIM mode with various in-source collision energies, 0 V, 20 V, and 40 V, respectively. The following parameters were used: mass scan range was set from 150 to 1000 m*/z* for CE = 0 V, 50 to 500 m*/z* for CE = 20 V and 40 V, resolution 70,000 for CE = 0 V, 35,000 for CE = 20 V and 40 V, AGC target of 5e5 and maximum IT of 100 ms for CE = 20 V, and 115 ms for CE = 40 V.

For data-dependent acquisition (DDA) mode, the samples were analyzed in negative ionization mode using the full-scan ddMS2 mode. Full-scan data was acquired with a resolution of 70,000, AGC target of 1e6, maximum IT of 100 ms, and scan range from 150 to 800 m*/z*. The dd-MS2 conditions were set as follows: resolution of 17,500, AGC target of 1e5, maximum IT of 50 ms, loop count 5, MSX count 1, TopN 5, isolation window 1.5 m*/z*, isolation offset 0 m*/z*, scan range from 50 to 500 m*/z* and NCE 15, 35, 50. The dd settings were as follows: minimum AGC target of 1e4, intensity threshold of 2e5, charge exclusion of 2–8, > 8, and dynamic exclusion of 3.0 s.

For targeted MS/MS analysis data acquisition, the samples were re-run with full-scan ddMS2 mode after data analysis to include the candidate ions.

### Data analysis for non-targeted screening of unknown phthalate metabolites

**Fig. 1:**
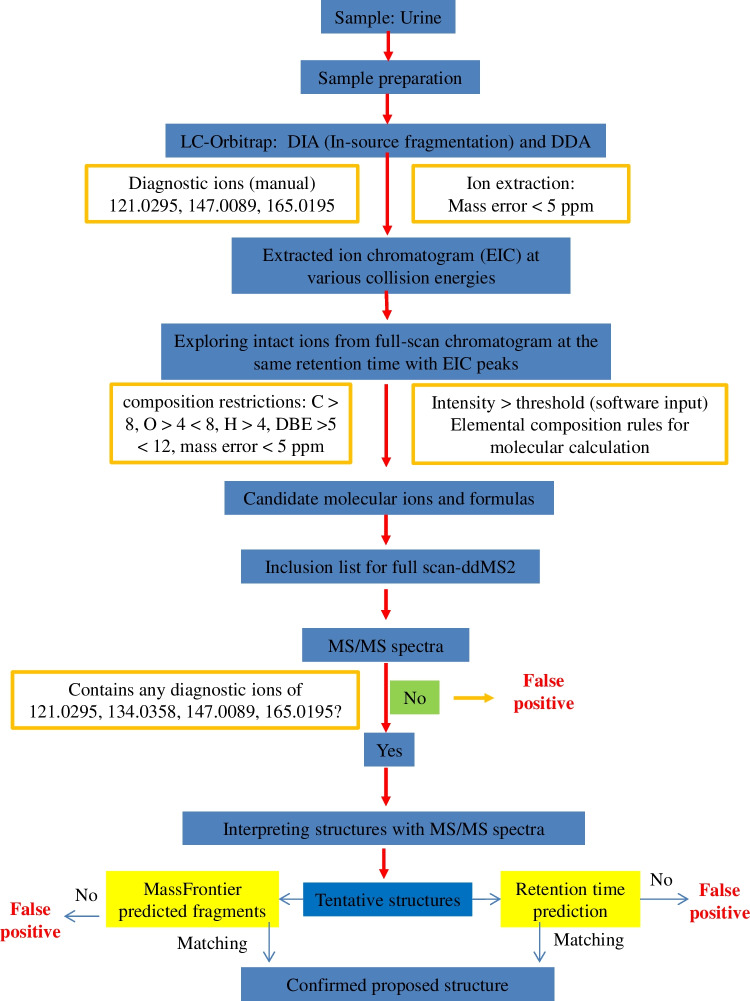
The workflow of non-targeted analysis approach

The workflow for data acquisition and data analysis is shown in Fig. 1. Thermo Fisher FreeStyle software (version 1.7) and Compound Discoverer (version 3.1.1) were used to perform the data analysis. We have previously demonstrated that most phthalate metabolites can usually produce a fragment ion at *m/z* 121.0295 and at least two out of three specific diagnostic fragment ions, *m/z* 121.0295 C_7_H_5_O_2_^−^, *m/z* 147.0089 C_8_H_3_O_3_^−^, and *m/z* 165.0193 C_8_H_5_O_4_^−^ [34]. Therefore, these three ions were used to generate extracted ion chromatograms (EICs) for both DIA data and DDA data to prioritize candidate molecular ions, using the FreeStyle Software. The fragment ion at *m/z* 134.0377 was used as a supplementary diagnostic ion to prioritize phthalate metabolites with pure alkyl chains as this is a specific product ion of this group of metabolites [34]. Molecules that do not produce these three specific ions are not shown in the EIC chromatogram and were filtered out. The precursor ions of DDA data were directly prioritized from EIC peaks with the following elemental composition restrictions for phthalate metabolites used for DIA data analysis. For DIA data, the peaks on the EICs of each specific ion at different collision energies were aligned and compared to the TIC at CE = 0 to identify the precise retention time of candidate precursor ions in the TIC. For both native and labelled compounds, an ion intensity threshold of 5 × 10^5^ counts in the TIC was entered manually in the peak extraction parameters within the FreeStyle software, which is five times higher than the signal-to-noise in spiked samples. Formulas were generated for ions with intensities higher than the threshold; molecular features for which formulas did not fall within elemental composition restrictions of phthalate metabolites were filtered out. A molecular feature can be defined as an entity for which mass, retention time, and intensity are assigned [19]. The ions that passed through this elemental restriction step were considered the potential candidate precursors in the inclusion list for targeted MS/MS analysis. The elemental composition restrictions for possible monophthalate features were carbon: 8–30; hydrogen: 6–60; oxygen: 4–10; no other elements; charge: − 1; nitrogen rule: even electron; relative double bond equivalent (RDBE): 6–12; mass tolerance: 5 ppm; S/N threshold: 3; minimum spectral fit: 10. Only those candidate precursor ions for which their MS/MS spectra contained at least two out of the three diagnostic ions were retained for further analysis, i.e., structure interpretation. Features for which the MS/MS spectra did not contain two out of the three diagnostic ions were considered false positives. Retention times were predicted for the proposed structures using the in-house retention time prediction model [35]. In this model, twenty-three molecular descriptors, representing the physicochemical properties of phthalates and their alternatives related to elution behavior such as partition-coefficient logP, were used, and its regression coefficient (*R*^2^) between predicted and experimental retention times was over 0.99 [35]. The predicted retention times were compared with the observed retention time as additional information to increase the identification confidence of structures interpreted from the MS/MS spectra.

Molecular features were also extracted using the Compound Discoverer software using the three replicates for blanks and pooled urine samples from fertile and infertile men. The data analysis workflow was untargeted metabolomics with statistics detecting unknowns with ID using local databases. Similar data processing parameters to FreeStyle feature extraction were used. Elemental composition was set as follows: minimum carbon 8, hydrogen 6, oxygen 4, and maximum carbon 30, hydrogen 60, and oxygen 10. Minimum abundance was set as 100 000, minimum RDBE 6, maximum RDBE 12, minimum S/N 3, mass tolerance 5 ppm, and accepted ions [M-H]^−^, and features present at least 3 times higher in urine samples compared to blanks were retained. The remaining parameters were left as default. Features were screened against the Thermo Fisher mzVault database and an in-house mass list containing 75 phthalate metabolites.

### QA/QC and method performance assessment

Thermo Fisher FreeStyle software was used for the data analysis in the method performance assessment. The instrumental detection limits were estimated as the lowest phthalate standard concentration for which a peak could be extracted and identified using at least 2 diagnostic ions (Table S2). Urine spiked with 100 ng of 24 native monophthalate standards was used to track the NTA performance in terms of repeatability by determining the relative standard deviation (RSD) of their signal intensities observed in triplicate and identification efficiency of the spiked standards (Table S2). For urine samples without native standards spiked, the labelled standards were added to monitor injections and to further check the efficiency of chemical identification. For positive identification in the pooled urine samples from infertile and fertile men, the intensity of the identified compounds (unknowns and labelled standards) had to be at least three times greater than that found in the corresponding procedural blank with a mass tolerance of 5 ppm.

## Results and discussion

### Influence of data acquisition methods on data analysis of monophthalate standard solutions

**Fig. 2:**
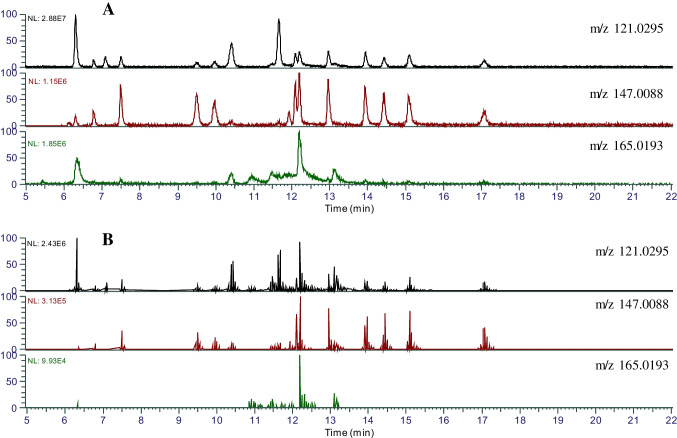
Extracted ion chromatograms (EIC). The concentration of monophthalates is 250 ppb. **A** EIC from DIA data, in-source full-scan, the in-source collision energy: 20 V; **B** EID from DDA data, full-scan ddMS2

**Fig. 3:**
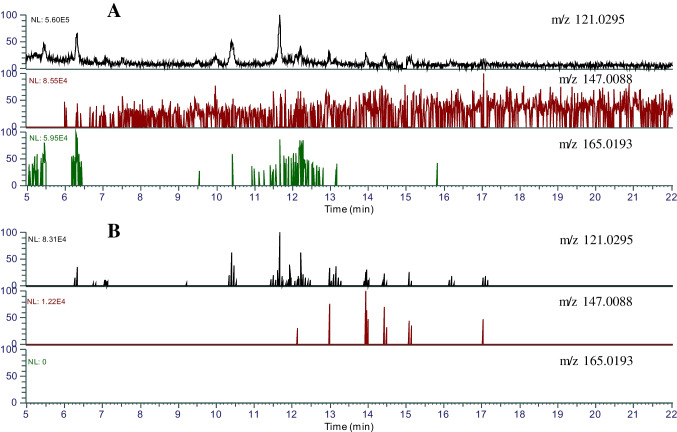
Extracted ion chromatograms (EIC). The concentration of monophthalates is 1 ppb. **A** EIC from DIA data, in-source full-scan, the in-source collision energy: 20 V; **B** EID from DDA data, full-scan ddMS2

In NTA, data acquisition and data analysis are two important factors that can influence the detectable chemical space thereby the final results. There are two common data acquisition methods in non-targeted analysis, the data-dependent acquisition (DDA) and data-independent acquisition (DIA). In DDA mode, the full-scan MS^2^ in Q-Orbitrap is the common data acquisition method. In this mode, the mass spectrometer automatically selects the precursor ions that meet the predefined ion abundance within a given *m/z* range in quadrupole MS 1 for performing MS/MS fragmentation in the collision cell and scan the product ions in Q-Orbitrap MS. In DIA mode, the full scan in Q-Orbitrap MS with in-source fragmentation is typically the common mode for data acquisition. Compared to DIA mode, DDA mode can increase the MS^2^ spectra quality due to less number of precursor ions being fragmented resulting in cleaner background [29]. In this study, two data acquisition modes were compared for their NTA performance on identification efficiency and accuracy. The previously developed three-diagnostic-ions method [34] was applied for analysis of the data acquired by both DIA and DDA, respectively. Figure 2 shows the difference between two extracted ion chromatograms (EIC) between DDA data and DIA data of 24 monophthalate standard solutions (250 ppb). The EIC from DIA data shows smooth EIC peaks extracted with three diagnostic ions, *m/z* 121.0295, *m/z* 147.0088, and *m/z* 165.0193. In contrast, the EIC from DDA data shows multiple MS/MS peaks at the retention times corresponding to the EIC peaks from DIA data. In our previous study, the retention times of EIC chromatographic peaks from DIA data were used to identify the precursor ions in the full scan data without collision energy and annotate the formulas for these precursor ions following the elemental composition restrictions [34]. Compared with the smooth EIC peaks from DIA data, the EIC MS/MS peaks from the DDA data can provide information on both retention time and precursor ions as the precursor ions can be directly identified from the EIC MS/MS peaks from DDA data. These precursor ions with the retention times identified from the EIC MS/MS peaks from DDA data can be used to identify their ion peak intensity in full scan data and calculate their molecular formulas under the element composition restrictions of phthalate metabolites. The results of this study also showed that EIC MS/MS peaks from DDA data are more selective than the EIC peaks from DIA data. Figure 3 shows that more peak groups can be observed in the EIC from the DDA data at two diagnostic ions, *m/z* 121.0295 and *m/z* 147.0088, with a good signal-to-noise ratio at 1 ppb (24 native monophthalate standards). However, at the same concentration level, only a few peaks can be observed on the EIC extracted from DIA data, and the peak signal-to-noise ratio is much lower than the EIC MS/MS peaks extracted from DDA data.

### Influence of data acquisition method on data analysis of unknowns in real samples

Compared with standard solutions, the sample matrix may affect the detectable chemical space and thereby the identification of unknowns in NTA. Therefore, it is of interest to investigate the difference between the two data acquisition methods and how each mode can influence the identification of unknown compounds in urine samples. We used the same data analysis protocol described above for screening of unknowns in the same urine samples and procedural water blanks spiked with 14 labelled monophthalate standards. Reproducibility was also assessed by determining the relative standard deviation (RSD) of spiked (100 ng) native monophthalate standards in urine samples, and RSD was satisfactory below 10%, except for monohexyl phthalate with an RSD of 10.4% (Table S2). We identified 86 precursor ions in the pooled urine (infertile men) and 89 precursor ions in the pooled urine (fertile men) with the DIA data as shown in Tables S3 and S4. FreeStyle molecular formula calculator was used to assign the formulas for all those precursor ions following the elemental composition restriction predefined for monophthalates. In contrast, with the DDA data, we identified 55 precursor ions in the pooled urine (infertile men) and 49 precursor ions in the pooled urine (fertile men), with molecular formulas also assigned following the same elemental composition restriction rule (Tables S5 and S6). For the identification of labelled monophthalates in samples, three diagnostic ions of *m/z* 124.0397, *m/z* 151.0223, and *m/z* 169.0329 were used to extract EIC peaks from both DDA and DIA data. Overall, 10 out of 14 spiked labelled standards were identified from the DIA data and 11 out of 14 labelled standards were identified from the DDA data. Although the total number of precursor ions extracted with the DDA data is less than the number of ions from the DIA data, the number of candidate ions from the DDA data, i.e., with more than one diagnostic ion, is higher compared to the DIA data (Table 1). This result shows that DDA data provided less false positive annotations although it has a lower hit rate compared to DIA data. Furthermore, NTA through DIA mode needs a separate acquisition of the MS/MS spectra for these precursor ions prioritized in the first step of EIC extraction with the three diagnostic ions and formula assignment with the elemental composition restriction. The DDA data also provided a better sensitivity in non-targeted screening compared to the DIA data as 11 of 14 labelled standards were identified from the DDA data (Tables S5 and S6) and only 10 were identified from the DIA data (Tables S3 and S4), likely due to more selectivity of DDA data by reducing background noise and improvement in the transfer of relevant fragment ions. Interestingly, using DIA data, there are 3 non-spiked labelled molecular features that were identified in the pooled urine (infertile men) which gives a false positive rate of 21.4%, while 2 labelled molecular features were identified in pooled urine from fertile men, with a false positive rate of 14.3%. Likewise, using DDA data, there is only 1 non-spiked labelled molecular feature identified in both pooled urine (infertile and fertile men), with a false positive rate of 7.1%. Therefore, the DDA data provided a lower false positive than the DIA data. Overall, the DDA data can provide clearer MS/MS spectra, which can improve the identification of monophthalates. In contrast, the precursor ions prioritized from DIA data have to be re-analyzed in MS/MS mode, to obtain their fragmentation spectra for further step identification.

**Table 1:** Number of ions detected and identified with different levels of confidence [36] in urine samples (from fertile and infertile men) using DDA and DIA acquisition modes

Acquisition mode	Precursor ions		Candidate ions		Level 1 identification		Level 2 identification		Level 3 identification	
	Fertile	Infertile	Fertile	Infertile	Fertile	Infertile	Fertile	Infertile	Fertile	Infertile
DIA	89	86	16	9	6	5	3	1	7	3
DDA	49	55	19	13	6	3	3	1	10	8

### Comparison of data analysis approaches

In NTA, various data analysis approaches can also lead to different results in prioritizing precursor ions and molecular features and identifying unknowns in the sample. In this study, we compared the three-diagnostic-ion method developed previously [34] with the Compound Discoverer software from Thermo Fisher Scientific. The same elemental composition restriction was set up to meet the structure of phthalate metabolites. However, the Compound Discoverer software is not able to extract peaks with the three diagnostic ions. Rather, features are annotated based on elemental composition or matches against databases or mass lists. A total of 3323 features were extracted, with 604 features annotated as known compounds matched through mzVault or the metabolite mass list. Within those 604 features, 132 features were annotated as possible phthalate metabolites (listed in Table S7), more than those observed using the diagnostic ion approach. However, some compounds were annotated as phthalate metabolites at more than one retention time; for example, mono-5-hydroxy hexyl phthalate was annotated as such at nine different retention times. Accounting for the replicate retention times, only 32 phthalate metabolites can be considered annotated (Table S7). These features were retained for further data analysis. Each of the annotated monophthalate features was manually screened using the MS2 information from Compound Discoverer to confirm the presence/absence of diagnostic ions in the ddMS2-acquired samples. A false positive rate of 21.2% was observed, with 28 out of 132 features identified that showed the presence of only one diagnostic ion. All annotated monophthalate metabolites identified through Compound Discoverer were also identified, based on mass and general formulas, as possible phthalate metabolites through the FreeStyle data analysis, except for mono-5-carboxypentyl, mono-5-oxohexyl, mono-4-hydroxy-pentyl, mono-6-hydroxy-heptyl, mono-4-methyl-7-oxooctyl, mono-7-hydroxy-4-methyloctyl, mono-3-carboxypropyl, monocyclohexyl, and monopentyl phthalate. However, as these features showed only one or no diagnostic ions from the MS2 spectra, they were identified as false positives in Compound Discoverer. Overall, using the Compound Discoverer, 114 out of 132 features annotated as possible phthalate metabolites (86%) were false positives, i.e., had none or only one diagnostic ion. A similar rate was observed for DIA data analyzed using FreeStyle software, where 150 out of 176 features across both samples from fertile and infertile men were considered false positives (85%). On the other hand, a lower rate (70%) was found for DDA data using FreeStyle, with 73 out of 104 features across samples from fertile and infertile men, only showing one or no diagnostic ions.

One molecular feature, at the retention of 12.7 min, with mass 322.1783 and *m/*z 321.1709, was identified as a false positive (based on the presence of only fragment ion *m/z* 121.0297) through Compound Discoverer as mono-2-propyl-6-hydroxyheptyl phthalate. This same feature (retention time 12.6 min) was identified with the FreeStyle diagnostic ion approach (Tables S3–S6); however, another diagnostic ion at *m/z* 147.0087 was detected through FreeStyle, but could not be observed in the Compound Discoverer. This feature was identified using FreeStyle, in both pooled urine samples (fertile and infertile men) using the DIA data, and only in the pooled urine sample from infertile men using the DDA data. The DIA full-scan SIM data cannot be used in the Compound Discoverer to evaluate MS/MS spectra in order to improve compound annotation, which is possibly why the *m/z* 147.0087 ion was not observed in Compound Discoverer. Similarly, no fragment ion at *m/z* 147.0087 was observed for mono-2-ethyl-5-oxohexyl in Compound Discoverer, while it was observed using the FreeStyle software. This metabolite was also only identified using DIA data in FreeStyle, and since only the DDA (ddMS2) data was used in the Compound Discoverer to inspect MS/MS spectra, it is possibly why the fragment is not seen.

Another molecular feature, identified as mono-2-ethyl-5-carboxypentyl, in Compound Discoverer, was annotated at retention times of 12.90 min and 10.96 min. Both features showed 2 diagnostic ions; however, the feature identified from the Compound Discoverer at the retention time of 10.96 min, in the pooled urine sample from fertile men, was not identified with the FreeStyle. Upon further inspection of the chromatogram, it could be seen that the intensity of the peak was around 10E3, therefore, possibly near the limit of detection and below the threshold set in FreeStyle (5E5).

Features annotated using Compound Discoverer are not limited to a specific group of compounds, and since online databases or mass lists can be included during data processing, the software can allow for a more comprehensive screening and annotation of compounds present. In contrast, the three-diagnostic-ion method can narrow down to specific molecular features, i.e., precursor ions that belong to groups of chemicals that have a similar structure skeleton that produce the same pattern of fragment ions. Although the feature annotation using the three-diagnostic-ion method is much smaller than the Compound Discoverer, the rate of false positives is lower, using DDA data. On the other hand, feature annotation in the Compound Discoverer is limited to compound information present in the databases used. For example, mono-3-propyl-4-oxohexyl was identified (level 2) using the diagnostic ion approach, but this compound is not present in mzVault or the in-house metabolite database; it was not annotated as such in the Compound Discoverer. Because data analysis using online or local databases, like the Compound Discoverer, is limited to the information contained in those databases, other data analysis and filtering approaches are needed to identify new compounds for which mass or spectral information is not yet available, and therefore covered by online databases. Upon inspection of the complete feature list (3323) identified through the Compound Discoverer, a feature corresponding to the formula, mass, and retention time of mono-3-propyl-4-oxohexyl (identified through FreeStyle) was identified. Both diagnostic fragment ions identified in FreeStyle, *m/z* 121.0296 and *m/z* 147.0094, were also observed in the ddMS2 spectra from the Compound Discoverer. The results in this study indicated using the diagnostic ion approach coupled to database screening can offer a more comprehensive coverage of phthalate metabolites and reduce the false positive identification of unknown compounds in NTA.

### The difference of phthalate metabolites between urine samples from fertile and infertile men with two data analysis approaches

**Fig. 4:**
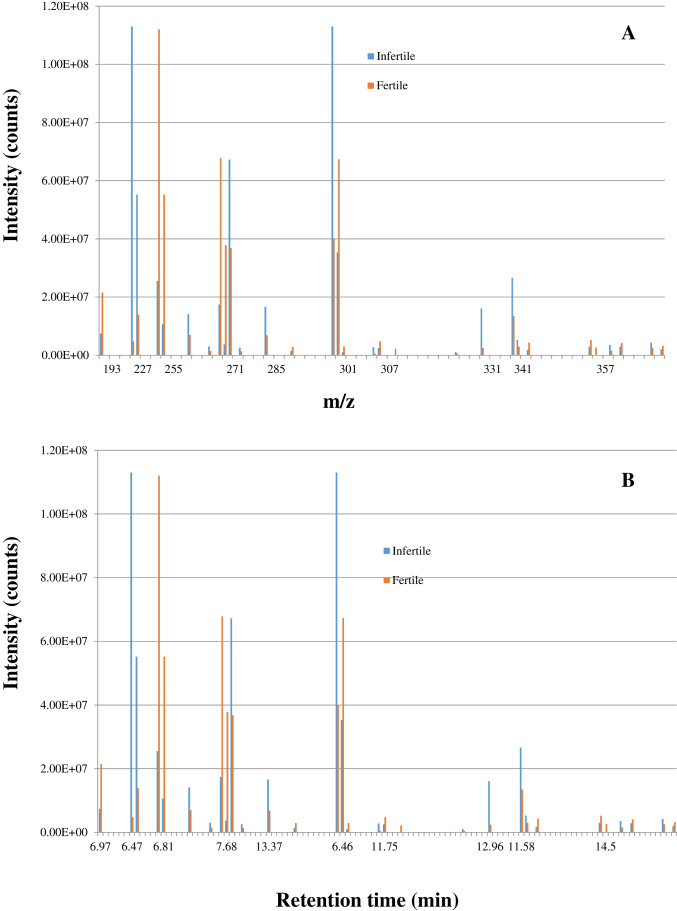
The difference of precursor ions between urine samples from infertile and fertile men. **A** the intensity difference of precursor ions between urine samples from infertile and fertile men; **B** the intensity difference of precursor ions at retention time between urine samples from infertile and fertile men

It is important for NTA to distinguish the difference between two groups of samples. In this study, we also compared the difference of identified precursor ions or molecular features between two samples pooled from two different groups of urines, both through the Compound Discoverer and three-diagnostic-ion approaches. Figure 4 shows the difference of precursor ions identified by the three-diagnostic-ion data analysis approach between two pooled urine samples. Figure 4A shows that the intensity of the compound (or molecular features) with *m/z* 227.035 at 6.5 min in the pooled urine (infertile men) is about 25 times higher than in the pooled urine (fertile men). While comparing the molecular features on the scale of retention time (Fig. 4B), the intensities of compounds (or molecular features) with *m/z* 263.129 at 6.85 min, *m/z* 265.0719 at 7.9 min, *m/z* 271.0613 at 8.04 min, *m/z* 275.0927 at 9.8 min, *m/z* 285.0772 at 13.4 min, *m/z* 321.171 at 12.2 min, *m/z* 341.1032 at 11.6 min, *m/z* 363.2182 at 15.2 min, and *m/z* 407.2077 at 12.1 min in the pooled urine (infertile men) is about two times higher than in the pooled urine (fertile men). The intensities of compounds (or molecular features) with *m/z* 301.0719 at 9.7 min, *m/z* 307.1553 at 10.6 min, and *m/z* 331.1919 at 12.96 min in the pooled urine (infertile men) are about three times, six times, and seven times higher than in the pooled urine (fertile men), respectively. This result demonstrated that the three-diagnostic-ion approach can profile the difference between a specific group of chemicals on a three-dimensional scale, retention time, intensity, and molecular ions.

**Fig. 5:**
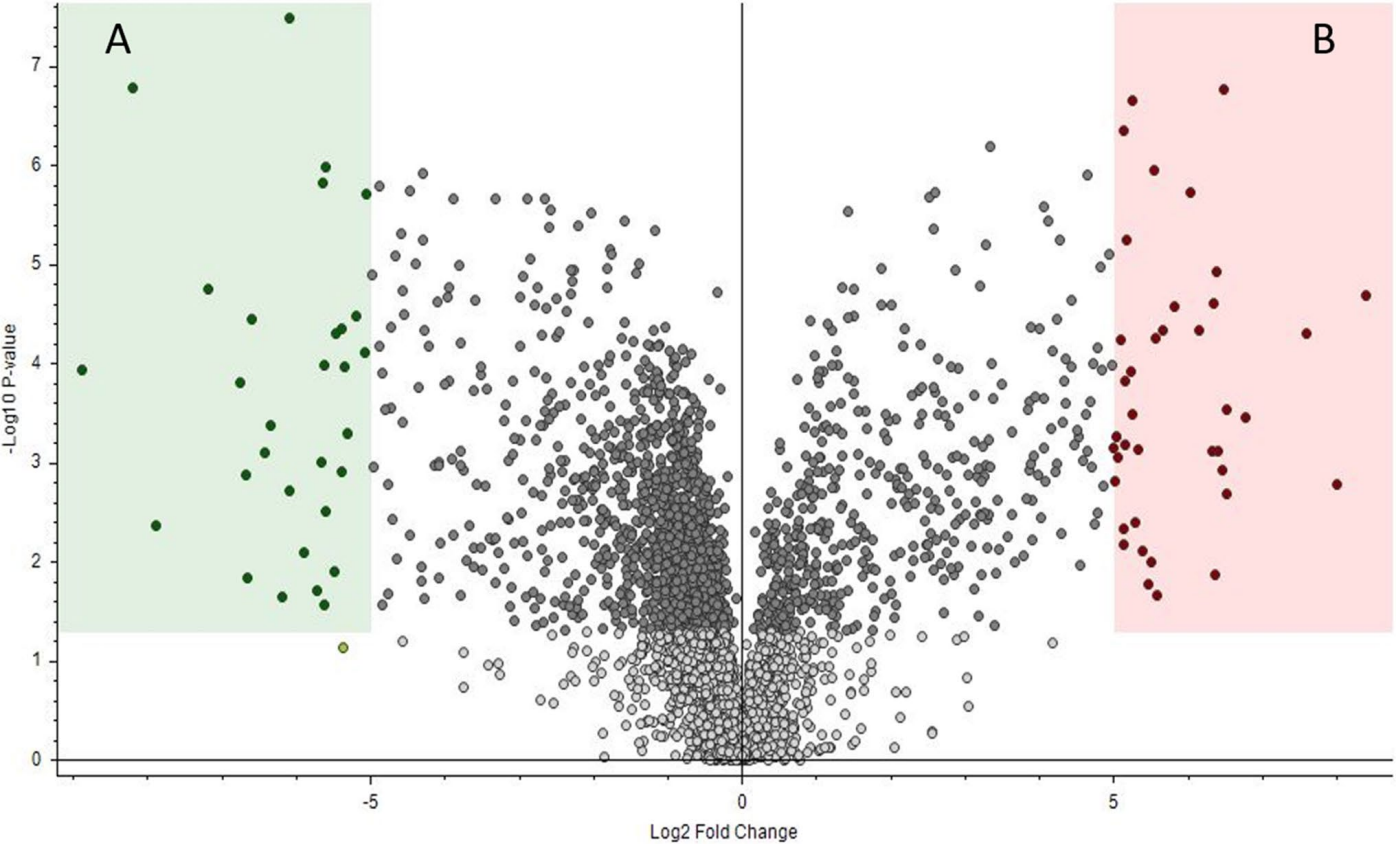
Molecular features discovered by Compound Discoverer between urine from fertile and infertile men with a log-fold change > 2, *p* < 0.05. **A** Downregulated features that are present with higher intensity in samples (fertile men); **B** upregulated features that are present with higher intensity in samples (infertile men)

Differential analysis can also be performed using the Compound Discoverer software. Figure 5 shows the features with a fold change > 2 (*p* < 0.05) when comparing urine samples from fertile and infertile men, with features downregulated in green and those upregulated in red. Checking the downregulated features on the graph automatically highlights the features in the feature table. Therefore, similar to the diagnostic ion approach, differential analysis using Compound Discoverer also shows the retention time, mass, and intensity of the statistically significant compounds.

### The identification of phthalate metabolites in two pooled urine samples

#### Data analysis: FreeStyle software

It was previously reported that most phthalate metabolites usually produce at least two of the three diagnostic ions, *m/z* 121.0295, *m/z* 147.0088, and *m/z* 165.0193 [34]. Therefore, those precursor ions that do not produce at least two of the diagnostic ions were considered false positive and excluded for structure interpretation/characterization in this study. Following this rule, the total numbers of precursor ions that can produce at least two of three diagnostic ions are 9 with the DIA data and 13 with the DDA data in the pooled urine (infertile men), respectively (Tables S3 and S5). The total numbers of precursor ions that can produce at least two of three diagnostic ions are 16 with the DIA data and 19 with the DDA data in the pooled urine (fertile men), respectively (Tables S4 and S6). Although the precursor ions identified with the DDA data are more than those identified with the DIA data, they overlap but do not fully cover each other. Using the MS/MS spectra, acquired using DIA data, and retention times of standards, 5 precursor ions (in urine collected from infertile men) and 6 precursor ions (in urine collected from fertile men) were identified as level 1 identification (Tables S3 and S4), according to the definition by Schymanski et al. [36]. Using the DDA data, only 3 and 6 precursor ions could be identified in urine samples collected from infertile and fertile men (Tables S5 and S6), at a level 1 identification. Overall, 10 precursor ions were identified at level 3 across the urine samples collected from fertile and infertile men using the DIA data (Tables S3 and S4), with 18 precursor ions identified from the DDA data across the two different samples (Tables S5 and S6). The retention time was predicted for the possible structures, using the developed in-house model [35], and was compared with the experimental retention time to improve the identification level for the structures elucidated by the MS/MS spectra to level 2 identification. Predicted retention times are listed in Tables S3 to S6. The difference between the predicted and observed retention times was less than 0.2 min for mono-7-methyloctyl phthalate, mono-3,4-dimethyl-5-ethyl-6-hydroxyhexyl phthalate, and mono-3-propyl-4-oxo-hexyl phthalate; therefore, the level of identification could be improved from level 3 to level 2. For the remaining features, the predicted retention times differed by more than 1 min, in some cases. For example, for *m/z* 321.1709, the predicted retention time for the proposed mono-6-methyl-7-hydroxynonyl phthalate is 13.63 min, which has over 1 min of retention time difference compared to their observed retention time at 12.66 min. Therefore, the identification confidence could not be improved from level 3 to level 2. For *m/z* 307.1189, the predicted retention time for proposed mono-1-hydroxy-2-oxo-5ethylhexyl phthalate is 12.07 min, which is about 0.5 min difference compared to the observed retention time at 12.66. Again, we leave it as level 3. With the aid of the retention time model, 1 out of 4 tentative structures in the pooled urine (infertile men) and 3 out of 10 tentative structures in the pooled urine (fertile men) were considered as the level 2 identification from DIA data (Tables S3 and S4). In contrast, 1 out of 8 tentative structures in the pooled urine (infertile men) and 3 out of 13 tentative structures in the pooled urine (fertile men) were considered level 2 identification from DDA data (Tables S5 and S6).

#### Data analysis: Compound discoverer

Mono-4-methyl-7-oxooctyl phthalate (retention time 12.58 min) was identified in the Compound Discoverer (Table S7) and showed the presence of 2 diagnostic ions, *m/z* 121.0296 and *m/z* 147.0087. This feature was identified in FreeStyle at two different retention times, 12.34 and 12.57 min (Table S4). Based on MassFrontier’s predicted fragmentation, isomers of mono-4-methyl-7-oxooctyl match the observed fragments in urine samples. Using complementary information, i.e., predicted retention time using the in-house prediction model [35], mono-4-methyl-7-oxooctyl has a predicted retention time of 11.90 min, while one of its isomers, mono-3-propyl-4-oxo-hexyl, had a predicted retention time of 12.57 min. Therefore, mono-3-propyl-4-oxo-hexyl was identified at the retention time of 12.57 min, level 2.

Mono-hydroxyisononyl phthalate was identified using Compound Discoverer, at retention times of 11.96 min and 12.97 min. The predicted retention time for this metabolite was 12.37 min. Based on predicted retention times for other isomers and generated fragments using MassFrontier, this feature was actually identified (level 2) as mono-3,4-dimethyl-5-ethyl-6-oxohexyl phthalate with a predicted retention time of 11.92 min, while it remained at level 3 identification at the retention time of 12.97 min, as an isomer of mono-hydroxynonyl phthalate. Mono-2-ethyl-5-carboxypentyl could not be improved from identification level 3 to 2 using the predicted retention time model; therefore, those features were identified as isomers. Mono-7-methyloctyl phthalate was identified as level 2 at a retention time of 15.74 min, with a predicted retention time of 15.58 min.

Interestingly, 7 molecular features were annotated as monoethyl phthalate. The retention time of monoethyl phthalate analytical standard is 6.78 min with one molecular feature, at the retention time of 6.63 min, integrated close to the retention time of the standard. All those seven features showed 2 diagnostic ions present at *m/z* 121.0296 and *m/z* 134.0373 and one molecular feature, at the retention time of 7.11 min, showing another ion at *m/z* 147.0087. However, many of those features were not identified in FreeStyle analysis, or they are identified as false positives. For example, the feature identified at the retention time of 5.55 min was not identified in FreeStyle, either using DIA or DDA data, although in Compound Discoverer it showed the presence of *m/z* 121.0296 and *m/z* 134.0373 ions. Two molecular features (Table S5 and S6) identified in samples from fertile and infertile men, using the DDA data, showed the same molecular formula, mass, and *m/z* as monoethyl phthalate. It is possible that those features, as well as the other features identified in the Compound Discoverer, correspond to isomers of monoethyl phthalate, such as 1-ethyl phthalic acid. We have observed that the reconstitution solvent can change the species of light monophthalate standards in the solution, which produces two separate peaks on chromatogram with the same mass and MS/MS spectrum (data is not shown). This might explain why multiple molecular features were observed and annotated as monoethyl phthalate. Nevertheless, this shows the challenges of NTA in the identification of real unknown unknowns.

Monobenzyl, monoheptyl, and mono-2-ethylhexyl phthalates were identified as level 1. Two features were identified as monobutyl phthalate, with retention times of 10.08 and 9.61 min, both showing two diagnostic ions. The first feature is indeed monobutyl phthalate, while the second feature is monisobutyl phthalate; both these metabolites were identified in FreeStyle. Overall, the data processing workflow in the Compound Discoverer allowed for the identification of 6, 9, and 4 features as levels 1, 2, and 3 respectively.

## Conclusion

To improve the reproducibility of NTA results, reduce false positives, and increase the identification confidence in non-targeted analysis, a harmonized strategy is necessary in developing and reporting NTA methods, such as data acquisition modes and data analysis approaches. The same data acquisition mode that was found to be more advantageous in this study, full-scan ddMS2, may not offer the same results in a different biological matrix, e.g., serum, or for a different chemical group, e.g., phenols. Overall, data acquisition modes have different advantages and disadvantages, and their influence on data analysis and interpretation must be systematically assessed and taken into account when developing NTA methods, for example, to determine the chemical space that can be described using a specific data acquisition mode. Understanding the influence of different data acquisition methods and different data analysis approaches on the reproducibility of NTA results is the key step to harmonize these strategies. The findings in this study indicate that the data acquisition method and data analysis method can affect the interpretation of NTA results. Therefore, it is necessary to develop a standard validation protocol when NTA results are reported. Different data acquisition methods and data analysis approaches may result in false positives. Data analysis of DIA data is more tedious, is limited to information included in databases, and still requires data acquisition in MS/MS mode to obtain fragment information and increase compound identification, while the data analysis of DDA data is more efficient and results in reduced false positives rates. Furthermore, data analysis workflows may also influence data interpretation. Therefore, it is crucial that data analysis/processing is included during the development of NTA workflows, besides sample preparation, where most of the focus has been so far. Both DDA and DIA data can be used to profile the difference between samples. The findings in this study may demonstrate NTA can identify a large number of chemicals for human exposure and its health effects.

## Supplementary Information

Below is the link to the electronic supplementary material.

Supplementary file1 (DOCX 154 KB)
