# Correction to: Influence of data acquisition modes and data analysis approaches on non‑targeted analysis of phthalate metabolites in human urine

**DOI:** 10.1007/s00216-022-04439-z

**Published:** 2022-11-22

**Authors:** Yong‑Lai Feng, Anca Baesu

**Affiliations:** Exposure and Biomonitoring Division, Environmental, Health Science and Research Bureau, Environmental and Radiation Health Sciences Directorate, Healthy Environments and Consumer Safety Branch, Health Canada, AL: 2203 B, 251 Sir Frederick Banting Driveway, Ottawa, ON K1A 0K9 Canada

Correction to: Analytical and Bioanalytical Chemistry

https://doi.org/10.1007/s00216-022-04407-7

The original article contained a mistake.

The copyright holder name should be presented as:

“© His Majesty the King in Right of Canada, as represented by the Minister of Health, 2022.” instead of, “@Crown 2022”

The original article has been corrected.

