# Supplementary material for: Influence of data acquisition modes and data analysis approaches on non-targeted analysis of phthalate metabolites in human urine
Source: Anal Bioanal Chem. 2022 Nov 8;415(2):303–16. doi: 10.1007/s00216-022-04407-7 (PMC9823047; doi:10.1007/s00216-022-04407-7)
Supplement: Supplementary file 1 — Supplementary file1 (DOCX 154 KB) [file 216_2022_4407_MOESM1_ESM.docx]

Table S1: Isotopic labelled monophthalate standards spiked in urine samples

| Name | Precursor ion (*m/z*) |
| --- | --- |
| Monomethyl phthalate (MMP, ring-1,2-^13^C_2_, dicarboxyl-^13^C_2_) | 183.048 |
| Monoethyl phthalate (MEP, ring-1,2-^13^C_2_, dicarboxyl-^13^C_2_) | 197.0637 |
| Mono-n-butyl phthalate (MBP, ring-1,2-^13^C_2_, dicarboxyl-^13^C_2_) | 225.095 |
| Monocyclohexyl phthalate (MCyHP, ring-1,2-^13^C_2_, dicarboyxl-^13^C_2_) | 251.1106 |
| Monobenzyl phthalate (MBzP, ring-1,2-^13^C_2_, dicarboxyl-^13^C_2_) | 259.0783 |
| Mono-n-octyl phthalate (MOP, ring-1,2-^13^C_2_, dicarboxyl-^13^C_2_) | 281.1574 |
| Mono-2-ethylhexyl phthalate (MEHP, ring-1,2-^13^C_2_, dicarboxyl-^13^C_2_) | 281.1574 |
| Mono-(7-methyloctyl) phthalate (7-MMOP, ring-1,2-^13^C_2_, dicarboxyl-^13^C_2_) | 295.1731 |
| Mono-3,7-dimethyl-1-octyl phthalate (3,7-diMMOP, ring-1,2-^13^C_2_, dicarboxyl-^13^C_2_) | 309.1888 |
| Mono-3-carboxypropyl phthalate (MCPP, ring-1,2-^13^C_2_, dicarboxyl-^13^C_2_) | 255.0692 |
| Mono-2-ethyl-5-carboxypentyl phthalate (MECPP, ring-1,2-^13^C_2_, dicarboxyl-^13^C_2_) | 311.1317 |
| Mono-2-(carboxymethyl) hexyl phthalate (MCMHP, ring-1,2-^13^C_2_, dicarboxyl-^13^C_2_) | 311.1317 |
| Mono-2-ethyl-5-oxohexyl phthalate (MEOHP, ring-1,2-^13^C_2_, dicarboxyl-^13^C_2_) | 295.1367 |
| Mono-2-ethyl-5-hydroxyhexyl phthalate (MEHHP, ring-1,2-^13^C_2_, dicarboxyl-^13^C_2_) | 297.1523 |

Table S2: Instrumental detection (IDL) and reproducibility (*n*=3, spiked at 100 ng) of 24 native standards

| Chemical name | *m/z* | Retention time (min) | IDL, ng/mL | | RSD |
| --- | --- | --- | --- | --- | --- |
|  |  |  | DIA | DDA |  |
| mono-3 hydroxybutyl phthalate (3-OH-MBP) | 237.0768 | 6.3 | 1 | 1 | 4.2 |
| mono-3-carboxypropyl phthalate (MCPP) | 251.0561 | 6.3 | 1 | 2 | 6.2 |
| Monomethyl phthalate (MMP) | 179.035 | 6.1 | 1 | 1 | 9.2 |
| monomethyl isopththalate (MMiP) | 179.035 | 7.1 | 1 | 1 | 9.1 |
| Monoethyl phthalate (MEP) | 193.0506 | 6.7 | 1 | 1 | 7.0 |
| Monoisopropyl phthalate (MiPP) | 207.0663 | 9.4 | 1 | 1 | 7.0 |
| mono-2-ethyl-5-carboxypentyl phthalate (MECPP) | 307.1187 | 10.9 | 1 | 1 | 6.9 |
| mono-7-carboxy-heptyl phthalate (MCHP) | 307.1187 | 11.5 | 1 | 1 | 4.6 |
| mono-2-ethyl-5-hydroxyhexyl phthalate (MEHHP) | 293.1394 | 10.4 | 1 | 1 | 6.2 |
| Monobutyl phthalate (MBP) | 221.0819 | 9.9 | 1 | 1 | 3.8 |
| Monoisobutyl phthalate (MiBP) | 221.0819 | 9.4 | 1 | 1 | 3.8 |
| mono-2-ethyl-5-oxohexyl phthalate (MEOHP) | 291.1238 | 11.6 | 1 | 1 | 3.9 |
| Monocarboxyisooctyl phthalate (MCiOP) | 321.1344 | 12.1 | 1 | 1 | 4.8 |
| mono-2-carboxy-methylhexyl phthalate (MCMHP) | 307.1187 | 11.8 | 1 | 1 | 7.9 |
| Monobenzyl phthalate (MBzP) | 255.0663 | 11.9 | 1 | 1 | 1.5 |
| Monocyclohexyl phthalate (MCyHP) | 247.0976 | 12.2 | 1 | 1 | 6.9 |
| Monopentyl phthalate (MPP) | 235.0979 | 12 | 1 | 1 | 2.5 |
| Monocarboxynonyl phthalate (MCNP) | 335.15 | 13.1 | 1 | 1 | 7.3 |
| Monohexyl phthalate (MHP) | 249.1132 | 12.9 | 1 | 1 | 10.4 |
| Monoheptyl phthalate (MHpP) | 263.1289 | 13.9 | 1 | 1 | 4.1 |
| mono-2-ethylhexyl phthalate (MEHP) | 277.1445 | 14.4 | 1 | 1 | 2.7 |
| Monooctyl phthalate (MOP) | 277.1445 | 15.1 | 1 | 1 | 4.1 |
| Monoisononyl phthalate (MiNP) | 291.1602 | 13.7 | 1 | 1 | 4.5 |
| Monoisodecyl phthalate (MiDP) | 305.1758 | 17.1 | 1 | 1 | 1.6 |

IDL is estimated as the lowest phthalate standard concentration for which a peak can be extracted and identified using at least two diagnostic ions

Table S3, List of precursor ions identified in pooled urine from infertile men with the DIA data

| *m/z* | Predicted RT (mini) | RT (min) | EIC ions | Identified compounds (Identification level) | Peak intensity (counts) | Major ions | Observed diagonstic ions | calculated formula | Theo Mass (m/z) | Difference (ppm) | RDB | Pattern Cov. (%) |
| --- | --- | --- | --- | --- | --- | --- | --- | --- | --- | --- | --- | --- |
| 293.1033 |  | 6.27 | 121.0295 | false positive | 5.09E+06 | 293.1031, 213.0922, 190.0272, 189.0194, 177.1285, 121.0296, 79.9574 | 121.0296 | C_15_H_17_O_6_ | 293.10306 | 0.82 | 7 | 100 |
| 191.035 |  | 6.26 | 121.0295 | false positive | 5.24E+06 | 176.0115, 148.0166, 104.0269 | 121.0296 | C_10_H_7_O_4_ | 191.03498 | 0.35 | 7 | 99.24 |
| 193.0506 |  | 6.16 | 121.0295 | false positive | 1.78E+06 | 193.0507, 178.0268, 161.0246, 149.0608, 137.0246, 134.0374, 121.0295, 93.0347 | 121.0295 | C_10_H_9_O_4_ | 193.05063 | -0.25 | 6 | 100 |
| 261.0769 |  | 6.23 | 121.0295 | false positive | 1.04E+06 | 181.0766, 179.0714, 137.0245, 136.0166, 93.0347 | 121.0288 | C_14_H_13_O_5_ | 261.07685 | 0.1 | 8 | 100 |
| 227.035 |  | 6.47 | 121.0295 | false positive | 1.13E+08 | 227.0351, 199.0402, 183.0451, 171.0452, 159.0452 | NIL | C_13_H_7_O_4_ | 227.03498 | 0.16 | 10 | 98.92 |
| 193.0506 |  | 6.51 | 121.0295 | false positive | 2.82E+06 | 193.0505, 149.0608, 134.0374, 121.0296 | 121.0296 | C_10_H_9_O_4_ | 193.05063 | -0.33 | 6 | 100 |
| 301.1446 |  | 6.46 | 121.0295 | false positive | 3.53E+07 | 301.1446, 271.1342, 253.1236, 241.1236, 121.0295, 107.0503 | 121.0295 | C_18_H_21_O_4_ | 301.14453 | 0.2 | 8 | 98.17 |
| 191.0351 |  | 6.77 | 121.0295, 147.0089 | false positive | 2.51E+06 | 191.0352, 176.0116, 117.0194, 111.0089, 85.0296 | 121.0296 | C_10_H_7_O_4_ | 191.03498 | 0.67 | 7 | 99.24 |
| **193.0506** |  | **6.77** | **121.0295, 147.0089** | **MEP^a^ (Level 1)** | **3.65E+06** | **193.0507, 149.0609, 121.0295,79.9574** | **121.0295, 147.0090** | **C_10_H_9_O_4_** | **193.05063** | **-0.02** | **6** | **99.24** |
| 227.0351 |  | 6.86 | 121.0295, 147.0089 | false positive | 5.52E+07 | 227.0352, 226.0273 | NIL | C_13_H_7_O_4_ | 227.03498 | 0.7 | 10 | 98.92 |
| 255.0665 |  | 6.81 | 121.0295, 147.0089 | false positive | 1.06E+07 | 149.0244, 135.0089, 121.0296, 91.0191 | 121.0296 | C_15_H_11_O_4_ | 255.06628 | 0.85 | 10 | 98.7 |
| 261.0769 |  | 6.8 | 121.0295, 147.0089 | false positive | 2.49E+06 | 261.0765, 217.0871, 201.0559, 199.0763, 189.0559, 181.0509, 173.0608, 161.0609, 135.0454, 96.9602, 79.9575, 69.0346, 59.0137 | 121.0295 | C_14_H_13_O_5_ | 261.07685 | 0.1 | 8 | 98.67 |
| 263.129 |  | 6.84 | 121.0295, 147.0089 | false positive | 1.41E+07 | 219.1392, 204.1158, 203.1079, 163.0768, 152.0846, 151.0767, 139.0765, 138.0689, 125.0608, 97.0295 | NIL | C_15_H_19_O_4_ | 263.12888 | 0.44 | 6 | 99.87 |
| 313.1085 |  | 6.86 | 121.0295, 147.0089 | false positive | 1.23E+07 | 269.1182, 147.0816, 121.0296, 109.0295 | 121.0296 | C_18_H_17_O_5_ | 313.10815 | 1.1 | 10 | 97.99 |
| 315.1603 |  | 6.83 | 121.0295, 147.0089 | false positive | 3.47E+06 | 315.1601, 235.0773, 163.0766, 149.0608, 147.0816, 131.0503, 121.0296 | 121.0296 | C_19_H_23_O_4_ | 315.16018 | 0.3 | 8 | 100 |
| 319.1916 |  | 6.84 | 121.0295, 147.0089 | false positive | 2.99E+07 | 319.1917, 275.2017, 273.1864, 83.0504 | NIL | C_19_H_27_O_4_ | 319.19148 | 0.33 | 6 | 100 |
| 193.0506 |  | 6.97 | 121.0295, 165.0193 | false positive | 7.38E+06 | 193.0507, 175.0400, 149.0609, 131.0502, 107.0503, 105.0709 | 121.0296 | C_10_H_9_O_4_ | 193.05063 | -0.1 | 6 | 99.24 |
| 207.0663 |  | 6.9 | 121.0295, 165.0193 | false positive | 1.16E+06 | 207.0664, 163.0766, 135.0453, 122.0373, 109.0294, 93.0711 | 121.0294 | C_11_H_11_O_4_ | 207.06628 | 0.16 | 6 | 100 |
| 249.1134 |  | 6.91 | 121.0295, 165.0193 | false positive | 6.38E+06 | 205.1234, 135.0453, 123.0452 | 121.0296 | C_14_H_17_O_4_ | 249.11323 | 0.56 | 6 | 100 |
| 299.1291 |  | 6.96 | 121.0295, 165.0193 | false positive | 2.37E+06 | 183.0122, 173.0607, 149.0609, 131.0503, 107.0503, 84.0217 | 121.0293 | C_18_H_19_O_4_ | 299.12888 | 0.59 | 9 | 98.17 |
| 347.1867 |  | 6.92 | 121.0295, 165.0193 | false positive | 1.72E+06 | 303.1969, 301.1814, 285.1860, 275.1151, 255.1760, 124.0075, 106.9809, 85.0296, 79.9574, 75.0088 | NIL | C_20_H_27_O_5_ | 347.1864 | 0.8 | 7 | 97.66 |
| 235.0612 |  | 7.03 | 121.0295 | false positive | 2.69E+06 | 191.0714, 176.0479, 161.0245 | NIL | C_12_H_11_O_5_ | 235.0612 | 0.22 | 7 | 87.42 |
| 253.0506 |  | 7.09 | 121.0295 | false positive | 2.55E+07 | 253.0509, 224.0482 | NIL | C_15_H_9_O_4_ | 253.05063 | 0.05 | 11 | 98.7 |
| 273.077 |  | 7 | 121.0295 | false positive | 2.93E+06 | 193.0870, 137.0245, 136.0166, 93.0347 | NIL | C_15_H_13_O_5_ | 273.07685 | 0.55 | 9 | 86.04 |
| 191.035 |  | 7.19 | 121.0295 | false positive | 1.86E+06 | 191.0354, 176.0116, 147.0452, 85.0296, 73.0295 | 121.0295 | C_10_H_7_O_4_ | 191.03498 | 0.27 | 7 | 99.24 |
| 313.1085 |  | 7.26 | 121.0295 | false positive | 1.06E+07 | 313.1096, 191.0713, 135.0454, 121.0296, 107.0503, 93.0347, 83.0139 | 121.0296 | C_18_H_17_O_5_ | 313.10815 | 1 | 10 | 99.79 |
| 281.103 |  | 7.47 | 147.0089 | false positive | 1.30E+06 | 237.1135, 201.0924, 151.1133, 123.0816, 96.9602, 57.0344 | NIL | C_14_H_17_O_6_ | 281.10306 | -0.23 | 6 | 85.31 |
| 191.0349 |  | 7.47 | 147.0089 | ***1,2 benzenedicarboxylic acid, monoethenyl ester (level 3)*** | 1.99E+06 | 191.0351, 176.0116, 148.0168 | 121.0294, 147.0087 | C_10_H_7_O_4_ | 191.03498 | -0.21 | 7 | 100 |
| 271.0614 |  | 7.68 | 121.0295, 165.0193 | ***isomers of mono-2-hydroxy-4-methyl benzoate phthalate (level 3)*** | 3.67E+06 | 191.0352, 165.0193, 137.0245 | 121.0295, 165.0193 | C_15_H_11_O_5_ | 271.0612 | 0.64 | 10 | 98.52 |
| 349.2023 |  | 7.6 | 121.0295, 165.0193 | false positive | 3.36E+06 | 349.2025, 269.0463, 83/0139, 79.9575 | 121.0292 | C_20_H_29_O_5_ | 349.20205 | 0.81 | 6 | 97.65 |
| 271.0613 |  | 8.04 | 165.0193 | false positive | 6.72E+07 | 151.0037, 119.0502, 107.0139, 93.0346, 83.0140, 65.0032 | 165.0194 | C_15_H_11_O_5_ | 271.0612 | 0.42 | 10 | 99.84 |
| 269.0457 |  | 8.38 | 121.0295 | false positive | 1.74E+07 | 269.0458, 133.0295, 87.0452 | NIL | C_15_H_9_O_5_ | 269.04555 | 0.74 | 11 | 98.54 |
| 375.1817 |  | 8.59 | 165.0193 | false positive | 2.84E+06 | 313.1813, 282.1260, 124.0075, 123.0818, 96.9603, 85.0296, 79.9575 | NIL | C_21_H_27_O_6_ | 375.18131 | 1.04 | 8 | 97.32 |
| 263.1289 |  | 8.72 | 121.0295 | false positive | 1.82E+06 | 219.1391, 204.1153, 151.0767, 150.0687, 149.0608, 136.0529 | NIL | C_15_H_19_O_4_ | 263.12888 | 0.21 | 6 | 100 |
| 297.113 |  | 8.76 | 121.0295 | false positive | 4.23E+08 | 297.1133, 189.0559, 145.0657, 133.0660, 131.0504, 121.0660, 119.0502, 107.0503 | 121.0299 | C_18_H_17_O_4_ | 297.11323 | -0.86 | 10 | 100 |
| 331.1921 |  | 8.81 | 121.0295 | false positive | 1.26E+06 | 331.1915, 273.1505, 219.1025, 207.1393, 57.0343 | NIL | C_20_H_27_O_4_ | 331.19148 | 1.89 | 7 | 82.22 |
| 343.1186 |  | 8.76 | 121.0295 | false positive | 6.84E+06 | 263.0464, 167.1079, 113.0245, 85.0296, 71.0139 | 121.0295 | C_19_H_19_O_6_ | 343.11871 | -0.35 | 10 | 97.69 |
| 221.082 |  | 9.47 | 165.0193 | false positive | 1.14E+06 | 221.0829, 190.9985, 177.0919, 141.0194, 123.0089, 113.0245, 79.9574 | 121.0298 | C_12_H_13_O_4_ | 221.08193 | 0.31 | 6 | 99.13 |
| 301.0719 |  | 9.68 | 121.0295 | false positive | 1.13E+08 | 301.0719, 242.0597, 201.0197, 196.0013, 164.0116, 151.0037, 136.0166, 134.0374, 108.0218, 107.0139 | NIL | C_16_H_13_O_6_ | 301.07176 | 0.46 | 10 | 99.79 |
| 275.0927 |  | 9.82 | 121.0295 | false positive | 2.51E+06 | 275.0921, 189.0194, 153.1116, 152.1082, 121.0294, 93.0346 | 121.0294 | C_15_H15O_5_ | 275.0925 | 0.78 | 8 | 98.49 |
| 361.2025 |  | 9.77 | 121.0295 | false positive | 9.73E+05 | 331.1916, 316.1694, 297.1501, 271.1714, 259.1709, 137.0243, 124.0070, 96/9601 | 121.0298 | C_21_H_29_O_5_ | 361.20205 | 1.21 | 7 | 97.47 |
| 331.1918 |  | 9.9 | 121.0295 | false positive | 4.06E+06 | 331.1931,221.1549, 220.1469, 149.0609, 85.0296, 75.0088 | 121.0296 | C_20_H_27_O_4_ | 331.19148 | 1.06 | 7 | 100 |
| 407.2079 |  | 9.86 | 121.0295 | false positive | 1.43E+07 | 331.1917, 149.0608, 125.0606 | NIL | C_22_H_31_O_7_ | 407.20753 | 0.88 | 7 | 99.61 |
| **221.082** |  | **9.96** | **121.0295, 147.0089, 165.0193** | **MBP^a^ (Level 1)** | **1.43E+06** | **205.1230, 177.0927, 164.0840, 151.0874, 141.0194, 123.0089, 113.0245, 71.0503** | **121.0295, 147.0089** | **C_12_H_13_O_4_** | **221.08193** | **0.31** | **6** | **99.14** |
| 357.1347 |  | 9.95 | 121.0295, 147.0089, 165.0193 | false positive | 1.36E+06 | 147.0456, 123.0450, 122.0373, 96.9602, 79.9674 | 121.0296 | C_20_H_21_O_6_ | 357.13436 | 1.05 | 10 | 100 |
| 327.1241 |  | 9.99 | 121.0295 | false positive | 1.97E+06 | 197.0277, 183.0120, 171.1028, 137.0972 | 121.0295 | C_19_H_19_O_5_ | 327.1238 | 0.79 | 10 | 97.85 |
| **293.14** |  | **10.37** | **121.0295, 147.0089** | **MEHHP^a^ (Level 1)** | **3.66E+06** | **143.1078, 121.0295** | **121.0295, 147.0096** | **C_16_H_21_O_5_** | **293.13945** | **1.73** | **6** | **98.15** |
| 407.2079 |  | 10.41 | 121.0295, 147.0089 | false positive | 2.31E+06 | 331.1924, 96.9602, 93.0347 | NIL | C_22_H_31_O_7_ | 407.20753 | 0.96 | 7 | 96.54 |
| 307.1553 |  | 10.59 | 121.0295, 165.0193 | false positive | 2.75E+06 | 171.1027, 153.0923, 121.0295, 111.0816, 108.0218 | 121.0295 | C_17_H_23_O_5_ | 307.1551 | 0.67 | 6 | 100 |
| 357.1347 |  | 10.63 | 121.0295, 165.0193 | false positive | 2.96E+06 | 168.0430, 136.0530, 121.0295, 83.0139 | 121.0295 | C_20_H_21_O_6_ | 357.13436 | 1.05 | 10 | 100 |
| 333.2074 |  | 10.88 | 121.0295 | false positive | 8.58E+07 | 333.2075, 136.0529, 122.0375 | NIL | C_20_H_29_O_4_ | 333.20713 | 0.89 | 6 | 99.78 |
| 319.1916 |  | 10.87 | 121.0295 | false positive | 1.04E+07 | 319.1917. 275/2021 | NIL | C_19_H_29_O_4_ | 319.19148 | 0.24 | 6 | 97.97 |
| 333.2075 |  | 11.44 | 121.0295 | false positive | 7.69E+06 | 333.2075 | NIL | C_20_H_29_O_4_ | 333.20713 | 1.16 | 6 | 80.3 |
| 341.1032 |  | 11.58 | 121.0295, 165.0193 | false positive | 2.66E+07 | 341.1038, 176.0479, 175.0401, 123.0452, 121.0295, 109.0296, 93.0346, 69.0346 | 121.0295 | C_19_H_17_O_6_ | 341.10306 | 0.44 | 11 | 99.73 |
| 293.1399 |  | 11.56 | 121.0295, 165.0193 | false positive | 6.31E+06 | 231.1392, 216.1156, 177.0922, 162.0687, 115.0401, 71.0503 | 121.0296 | C_16_H_21_O_5_ | 293.13945 | 1.42 | 6 | 98.33 |
| 307.1553 | 11.92 | 11.77 | 121.0295, 165.0193 | ***mono-3,4-dimethyl-5-ethyl-6-hydroxyhexyl phthalate (level 2)*** | 2.49E+06 | 121.0295 | 121.0295, 147.0086, 165.0192 | C_17_H_23_O_5_ | 307.1551 | 0.77 | 6 | 100 |
| **291.1241** |  | **11.65** | **121.0295, 165.0193** | **MEOHP^a^ (Level 1)** | **2.39E+06** | **143.1079, 121.0295, 113.0974, 99.0817, 71.0502** | **121.0295, 147.0090** | **C_16_H_19_O_5_** | **291.1238** | **1.2** | **7** | **98.35** |
| 349.2023 |  | 11.61 | 121.0295 | false positive | 2.75E+06 | 319.1917, 269.1549, 79.9575 | NIL | C_20_H_29_O_5_ | 349.20205 | 0.73 | 6 | 97.64 |
| 265.1082 |  | 12.14 | 121.0295, 165.0193 | false positive | 1.25E+06 | 221.1285, 177.1285, 123.0813, 107.0503, 96.9600, 78.9591 | NIL | C_14_H_17_O_5_ | 265.10815 | 0.37 | 6 | 98.65 |
| 291.1604 |  | 12.17 | 121.0295, 165.0193 | false positive | 7.67E+06 | 291.1605, 247.1704, 246.1629, 148.0531, 135.0452, 122.0374 | 121.0294 | C_17_H_23_O_4_ | 291.16018 | 0.85 | 6 | 98.3 |
| 341.1032 |  | 12.17 | 121.0295, 165.0193 | false positive | 5.28E+06 | 341.1038, 176.0479, 123.0452, 121,0296, 109.0296, 93.0346, 69.0346 | 121.0296 | C_19_H_17_O_6_ | 341.10306 | 0.35 | 11 | 100 |
| 321.171 |  | 12.2 | 121.0295, 165.0193 | false positive | 1.02E+06 | 285.2069, 275.2025, 173.1184, 155.0714, 121.0296, 96.9602, 57.0343 | 121.0296 | C_18_H_25_O_5_ | 321.17075 | 0.76 | 6 | 98 |
| 349.2023 |  | 12.22 | 121.0295, 165.0193 | false positive | 1.39E+06 | 349.2023, 331.1920, 303.1970, 269.0458, 219.1394, 96.9601 | NIL | C_20_H_29_O_5_ | 349.20205 | 0.73 | 6 | 97.65 |
| 351.1816 |  | 12.36 | 121.0295 | false positive | 5.56E+06 | 336.1581, 321.1345, 319.1551, 305.1400, 275.1660, 260.1420, 182.0584, 167.0351, 152.0481, 151.0402 | NIL | C_19_H_27_O_6_ | 351.18131 | 0.94 | 6 | 97.67 |
| 393.2286 |  | 12.59 | 121.0295 | false positive | 9.92E+06 | 214.9749, 175.6680, 143.9718, 126.7275, 114.5681 | NIL | C_22_H_33_O_6_ | 393.22826 | 0.8 | 6 | 99.65 |
| 263.1291 |  | 12.67 | 121.0295 165.0193 | false positive | 1.03E+07 | 233.0821, 218.0586, 215.0714, 205.0871, 187.0765, 178.0274, 166.0268, 165.0196, 159.0817 | 165.0196 | C_15_H_19_O_4_ | 263.12888 | 0.79 | 6 | 98.65 |
| 307.1189 |  | 12.66 | 121.0295 165.0193 | false positive | 4.07E+07 | 233.0820, 219.0664, 218.0585, 215.0714, 187.0765, 165.0193, 137.0244 | 165.0193 | C_16_H_19_O_6_ | 307.11871 | 0.5 | 7 | 99.79 |
| 289.1077 |  | 12.66 | 121.0295 165.0193 | false positive | 3.29E+06 | 259.0612, 245.1155, 229.0871, 215.0714, 214.0637, 191.1052, 187.0766 | NIL | C_16_H_17_O_5_ | 289.10815 | -1.45 | 8 | 100 |
| 321.1709 |  | 12.67 | 121.0295 165.0193 | ***isomers of mono-6-methyl-7-hydroxy-nonyl phthalate (Level 3)*** | 1.27E+06 | 148.0528, 121.0296, 57.0344 | 121.0296, 147.0084 | C_18_H_25_O_5_ | 321.17075 | 0.38 | 6 | 98 |
| 359.1867 |  | 12.67 | 121.0295 165.0193 | false positive | 1.10E+06 | 315.1954, 299.1658, 287.2018, 285.1862, 150.0323, 123.0818, 96.9601 | NIL | C_21_H_27_O_5_ | 359.1864 | 0.95 | 8 | 97.48 |
| 317.1761 |  | 12.7 | 121.0295 165.0193 | false positive | 2.65E+06 | 317.1765, 299.1660, 273.1864, 135.0452, 123.0452, 122.0374 | NIL | C_19_H_25_O_4_ | 317.17583 | 0.99 | 7 | 97.79 |
| 377.2336 |  | 12.7 | 121.0295 165.0193 | false positive | 1.35E+06 | 377.2364, 341.1050, 297.1147, 269.2133, 253.1241, 191.0350, 176.0483, 96.9603 | 121.0295 | C_22_H_33_O_5_ | 377.23335 | 0.62 | 6 | 97.28 |
| 333.2075 |  | 12.98 | 121.0295 | false positive | 7.80E+07 | 333.2074, 144.0065, 137.0972, 119.0865, 112.0166, 55.0187 | NIL | C_20_H_29_O_4_ | 333.20713 | 1.07 | 6 | 100 |
| 255.0666 |  | 12.93 | 121.0295 | false positive | 4.52E+06 | 255.0666, 213.0559, 193.1597, 152.0037, 107.0140, 96.9602, 74.0248 | NIL | C_15_H_11_O_4_ | 255.06628 | 1.2 | 10 | 100 |
| 305.1761 |  | 12.94 | 121.0295 | false positive | 8.51E+06 | 305.1758, 261.1861, 149.0608, 136.0530, 122.0374 | 121.0296 | C_18_H_25_O_4_ | 395.17583 | 0.93 | 6 | 99.82 |
| 331.1919 |  | 12.97 | 121.0295 | false positive | 1.61E+07 | 331.1918, 287.2001, 269.1911, 191.1441, 83.0503 | NIL | C_20_H_27_O_4_ | 331.19148 | 1.34 | 7 | 97.79 |
| 285.0772 |  | 13.37 | 121.0295 | false positive | 1.66E+07 | 285.0770, 243.0663, 196.0014, 164.0116, 151.0037, 136.0166, 107.0140, 83.0139 | NIL | C_16_H_13_O_5_ | 285.07685 | 1.27 | 10 | 100 |
| 335.1869 |  | 13.4 | 121.0295 | false positive | 1.96E+07 | 177.0922, 162.0687 | NIL | C_19_H_27_O_5_ | 335.1864 | 1.56 | 6 | 99.76 |
| 347.1868 |  | 13.4 | 121.0295 | false positive | 1.06E+06 | 347.1866, 329.1769, 303.1966, 259.2072, 144.0065, 137.0967, 112.0170, 99.0449, 71.0502 | NIL | C_20_H_27_O_5_ | 347.1864 | 1.15 | 7 | 100 |
| 461.2914 |  | 14.43 | 121.0295, 147.0089, 134.0377 | false positive | 2.02E+06 | 461.2922, 443.2827, 431.2821, 413.2708, 295.2586, 162.8395, 160.8424, 123.0817, 96.9602 | NIL | C_27_H_41_O_6_ | 461.29086 | 1.06 | 7 | 99.49 |
| **277.1448** |  | **14.41** | **121.0295, 147.0089** | **MEHP^a^ (Level 1)** | **2.89E+06** | **147.0093, 134.0374, 127.1129, 121.0296, 75.0240** | **121.0296, 147.0093** | **C_16_H_21_O_4_** | **277.14453** | **1.1** | **6** | **98.5** |
| 363.2182 |  | 15.19 | 121.0295 | false positive | 3.51E+06 | 177.0921, 162.0687, 150.0685 | NIL | C_21_H_31_O_5_ | 363.2177 | 1.47 | 6 | 97.45 |
| 385.2389 |  | 15.36 | 121.0295 | false positive | 1.10E+07 | 385.2388, 367.2281, 341.2485, 339.2322, 323.2382, 215.1440, 123.0815, 57.0344 | NIL | C_24_H_33_O_4_ | 385.23843 | 1.19 | 8 | 99.67 |
| 349.1658 |  | 15.85 | 165.0193 | false positive | 2.98E+07 | 275.1294, 220.0742, 180.0429, 167.0350, 166.0272, | 1,650,191 | C_19_H_25_O_6_ | 349.16566 | 0.31 | 7 | 100 |
| 329.176 |  | 15.91 | 165.0193 | false positive | 2.66E+06 | 329.1761, 285.1860, 257.1911, 255.1754, 185.1334, 131.0866, 123.0816 | NIL | C_20_H_25_O_4_ | 329.17583 | 0.49 | 8 | 97.77 |
| 375.1815 |  | 18.64 | 165.0193 | false positive | 1.00E+07 | 301.1445, 233.0818, 219.0665, 218.0585, 204.0427, 201.0557, 191.0712, 167.0352, 137.0247 | 165.0195 | C_21_H_27_O_6_ | 375.18131 | 0.39 | 8 | 100 |
| *197.0641* |  | 6.78 | *124.0396* | *MEP (ring-1,2-^13^C_2_, dicarboxyl-^13^C_2_)* | 1.64E+06 | 162.8392, 160.8421, 137.0246, 124.0396 | 124.0396, 151.0222 | C_6_^13^C_4_H_9_O_4_ | 197.0637 | 2.02 | 6 | 0 |
| *225.0954* |  | 9.95 | *124.0396, 151.0220, 169.0328* | *MBP (ring-1,2-^13^C_2_, dicarboxyl-^13^C_2_)* | 4.79E+06 | 151.0219, 137.0473, 124.0395, 95.0414, 72.0536, 71.0502, 70.0379, 69.0345 | 124.0395, 151.0219 | C_8_^13^C_4_H_13_O_4_ | 225.09535 | 0.21 | 6 | 99.14 |
| *297.153* |  | 10.37 | *124.0396, 151.0220, 169.0328* | *MEHHP (ring-1,2-^13^C_2_, dicarboxyl-^13^C_2_)* | 1.51E+07 | 145.1235, 143.1078, 124.0396 | 124.0396, 151.0222 | C_12_^13^C_4_H_21_O_5_ | 297.15287 | 0.5 | 6 | 99.82 |
| 311.1325 |  | 10.85 | *124.0396, 169.0328* | *MCMHP (ring-1,2-^13^C_2_, dicarboxyl-^13^C_2_)* | 9.42E+05 | 237.1860, 159.1027, 124.0396, 113.0973 | 124.0396, 169.0327 | C_12_^13^C_4_H_19_O_6_ | 311.13213 | 1.11 | 7 | 85.25 |
| *295.1373* |  | 11.64 | *124.0396, 151.0220, 169.0328* | *MEOHP (ring-1,2-^13^C_2_, dicarboxyl-^13^C_2_)* | 2.13E+07 | 143.1078, 124.0396, 113.0972, 99.0816, 71.0502 | 124.0396, 151.0223 | C_12_^13^C_4_H_19_O_5_ | 295.13722 | 0.18 | 7 | 98.15 |
| *251.1111* |  | 12.2 | *124.0396, 151.0220, 169.0328* | *MCyHP (ring-1,2-^13^C_2_, dicarboyxl-^13^C_2_)* | 3.56E+07 | 151.0222, 125.0396, 97.0660, 95.0504 | 124.0396, 151.0222, 169.0328 | C_10_^13^C_4_H_15_O_4_ | 251.111 | 0.57 | 7 | 0 |
| *281.158* |  | 14.41 | *124.0396, 151.0220, 169.0328* | *MEHP (ring-1,2-^13^C_2_, dicarboxyl-^13^C_2_)* | 6.07E+07 | 151.0223, 137.0474, 127.1129, 124.0396, 77.0308 | 124.0396, 151.0223 | C_12_^13^C_4_H_21_O_4_ | 281.15795 | 0.03 | 6 | 98.34 |
| *311.1689* |  | 14.66 | *124.0396, 151.0220, 169.0328* | non-spiked | 3.95E+07 | 311.1689, 197.0279, 183.0121 | NIL | C_13_^13^C_4_H_23_O_5_ | 311.16852 | 1.28 | 6 | 0 |
| *295.1736* |  | 14.94 | *124.0396, 151.0220, 169.0328* | *7-MMOP (ring-1,2-^13^C_2_, dicarboxyl-^13^C_2_)* | 5.46E+07 | 295.1747, 151.0222, 142.1319, 141.1286, 139.1129, 137.0474, 124.0396, 69.0346 | 124.0396, 151.0222 | C_13_^13^C_4_H_23_O_4_ | 295.1736 | 0.04 | 6 | 0 |
| *281.1579* |  | 15.07 | *124.0396, 151.0220, 169.0328* | *(MOP) (ring-1,2-^13^C_2_, dicarboxyl-^13^C_2_)* | 5.81E+07 | 151.0223, 137.0474, 128.1163, 127.1129, 124.0396, 77.0309 | 124.0396, 151.0223 | C_12_^13^C_4_H_21_O_4_ | 281.15795 | -0.19 | 6 | 1.52 |
| *351.1638* |  | 15.04 | *124.0396, 151.0220, 169.0328* | non-spiked | 1.72E+06 | 351.1637, 96.9602 | NIL | C_15_^13^C_4_H_23_O_6_ | 351.16343 | 1.02 | 8 | 0 |
| *309.1892* |  | 17.05 | 124.0396, 151.0220, 169.0328 | *3,7-diMMOP (ring-1,2-^13^C_2_, dicarboxyl-^13^C_2_)* | 1.36E+08 | 156.1473, 155.1443, 154.1320, 153.1284, 151.0224, 137.0477, 124.0396, 77.0307 | 124.0396, 151.0224 | C_14_^13^C_4_H_25_O_4_ | 309.18925 | -0.24 | 6 | 0 |
| *339.2* |  | 17.11 | *124.0396, 151.0220, 169.0328* | non-spiked | 1.54E+06 | no | NIL | C_15_^13^C_4_H_27_O_5_ | 339.19982 | 0.49 | 6 | 0 |

Peak filter was set up as relative height of no less than 10 % of largest peak

*Italicized names and red color*: isotopically labelled compound

^a^: Confirmed with authentic standards (highlighted with orange)

^b^:Not confirmed with standards (highlighted with green)

Highlighted in yellow color are non-spiked labelled compounds

Table S4, List of precursor ions identified in pooled urine (fertile men) with the DIA data

| m/z | Predicted RT (min) | RT (min) | EIC ions | Identified compounds (Identification level) | Peak intensity | Major ions | Observed diagonstic ions | Calculated formula | Theo Mass (m/z) | Difference (ppm) | RDB | Pattern Cov. (%) |
| --- | --- | --- | --- | --- | --- | --- | --- | --- | --- | --- | --- | --- |
| 191.0351 |  | 6.27 | 121.0295 | false positive | 8.70E+06 | 191.0354, 161.0244, 147.0453, 85.0295, 79.9574 | 121.0296 | C_10_H_7_O_4_ | 191.03498 | 0.43 | 7 | 99.24 |
| 261.0769 |  | 6.23 | 121.0295 | false positive | 1.65E+06 | 181.0506, 166.0636, 164.0480, 137.0972, 125.0972, 79.9575 | 121.0297 | C_14_H_13_O_5_ | 261.07685 | 0.1 | 8 | 98.67 |
| 265.0718 |  | 6.22 | 121.0295 | false positive | 1.58E+06 | 185.0609, 96.9602 | 121.0301 | C_13_H_13_O_6_ | 265.07176 | 0.18 | 7 | 98.57 |
| 263.1289 |  | 6.33 | 121.0295 | false positive | 6.06E+06 | 245.0824, 183.0482, 175.0400, 168.0251, 145.0619, 127.0513, 79.9574 | NIL | C_15_H_19_O_4_ | 263.12888 | 0.21 | 6 | 84.77 |
| 193.0505 |  | 6.5 | 121.0295, 134.0377 | false positive | 3.72E+06 | 193.0506, 178.0636, 149.0608, 137.0608, 134.0375, 121.0295, 93.0347, 79.9575 | 121.0295 | C_10_H_9_O_4_ | 193.05063 | -0.65 | 6 | 100 |
| 301.1445 |  | 6.46 | 121.0295, 134.0377 | false positive | 6.73E+07 | 253.1239, 240.9898, 221.0281, 206.0588, 177.0922, 131.0378, 101.0067, 93.0347, 79.9574 | 121.0294 | C_18_H_21_O_4_ | 301.14453 | 0 | 8 | 100 |
| 263.129 |  | 6.69 | 121.0295 | false positive | 2.60E+06 | 219.1396, 204.1152, 203.1077, 183.0452, 145.0619, 127.0513, 109.0405, 79.9575 | 121.0298 | C_15_H_19_O_4_ | 263.12888 | 0.21 | 6 | 98.67 |
| 191.035 |  | 6.75 | 121.0295 | false positive | 1.93E+06 | 191.0355, 176.0116, 163.0404, 148.0167, 147.0453, 104.0269, 93.0346, 85.0296 | 121.0296 | C_10_H_7_O_4_ | 191.03498 | -0.05 | 7 | 100 |
| 193.0506 |  | 6.76 | 121.0295 | false positive | 2.24E+06 | 193.0507, 149.0608, 134.0374, 133.0295 | 121.0297 |  |  |  |  |  |
| 255.0664 |  | 6.82 | 121.0295, 147.0089 | false positive | 5.52E+07 | 149.0244, 135.0089, 121.0296, 91.0190 | 121.0296 | C_15_H_11_O_4_ | 255.06628 | 0.61 | 10 | 99.87 |
| 319.1915 |  | 6.85 | 121.0295, 147.0089 | false positive | 4.03E+07 | 319.1917, 275.2018, 83.0504 | NIL | C_19_H_27_O_4_ | 319.19148 | 0.14 | 6 | 99.8 |
| 263.1289 |  | 6.85 | 121.0295, 147.0089 | false positive | 6.96E+06 | 219.1391, 204.1156, 201.1287, 151.0765, 145.0620, 139.0766, 125.0609 | NIL | C_15_H_19_O_4_ | 263.12888 | -0.03 | 6 | 98.67 |
| 261.0769 |  | 6.82 | 121.0295, 147.0089 | false positive | 3.50E+06 | 261.0770, 217.0871, 203.0352, 201.0557, 199.0767, 189.0560, 181.0509, 173.0608, 161.0610, 147.0451, 135.0455, 69.0346, 59.0137 | 121.0294 | C_14_H_13_O_5_ | 261.07685 | 0.22 | 8 | 100 |
| **193.0506** |  | **6.98** | **121.0295, 165.0193** | **MEP^a^ (Level 1)** | **2.15E+07** | **193.0507, 178.0638, 175.0763, 149.0608, 134.0374, 123.0452, 121.0295, 93.0347** | **121.0295, 147.0084** | **C_10_H_9_O_4_** | **193.05063** | **-0.33** | **6** | **100** |
| 207.0662 |  | 6.92 | 121.0295, 165.0193 | false positive | 1.52E+06 | 207.0665, 163.0766, 122.0374, 93.0709 | 121.0297 | C_11_H_11_O_4_ | 207.06628 | -0.14 | 6 | 99.2 |
| 227.0349 |  | 6.86 | 121.0295, 165.0193 | false positive | 1.39E+07 | 227.0356, 147.0816, 79.9574 | NIL | C_13_H_7_O_4_ | 227.03498 | -0.44 | 10 | 99.03 |
| 249.1133 |  | 6.93 | 121.0295, 165.0193 | false positive | 5.34E+06 | 249.1137, 228.9895, 205.1234, 187.1130, 169.0656, 152.0843, 141.0922, 123.0816, 111.0452, 79.9574 | NIL | C_14_H_17_O_4_ | 249.11323 | 0.2 | 6 | 100 |
| 299.129 |  | 6.97 | 121.0295, 165.0193 | false positive | 2.69E+06 | 299.1305, 278.9853, 284.0553, 219.1029, 183.0121, 173.0608, 149.0608, 135.0454, 131.0504, 107.0504 | 121.0295 | C_18_H_19_O_4_ | 299.12888 | 0.28 | 9 | 98.17 |
| 313.1084 |  | 6.86 | 121.0295, 165.0193 | false positive | 1.00E+07 | 313.1083, 269.1180, 205.0505, 161.0610, 137.0608, 107.0503, 95.0503 | 121.0296 | C_18_H_17_O_5_ | 313.10815 | 0.8 | 10 | 98.01 |
| 315.1602 |  | 6.83 | 121.0295, 165.0193 | false positive | 3.60E+06 | 315.1610, 163.0766, 149.0609, 147.0816, 131.0503, 121.0295, 96.9602 | 121.0295 | C_19_H_23_O_4_ | 315.16018 | 0.01 | 8 | 97.99 |
| 347.1866 |  | 6.93 | 121.0295, 165.0193 | false positive | 4.30E+06 | 347.1870, 306.9807, 303.1965, 301.1814, 285.1859, 273.1870, 255.1754, 240.9893, 229.0682, 157.1234, 99.0089, 96.9601, 79.9575 | 121.0301 | C_20_H_27_O_5_ | 347.1864 | 0.72 | 7 | 97.64 |
| 235.0612 |  | 7.03 | 121.0295 | false positive | 3.10E+06 | 171.0817, 117.0752, 116.0717, 79.9675 | NIL | C_12_H_11_O_5_ | 235.0612 | 0.16 | 7 | 98.92 |
| 253.0507 |  | 7.08 | 121.0295 | false positive | 1.12E+08 | 253.0508, 96.9602, 79.9575, 59.0137 | NIL | C_15_H_9_O_4_ | 253.05063 | 0.17 | 11 | 100 |
| 191.035 |  | 7.18 | 121.0295 | false positive | 3.01E+06 | 191.0355, 176.0116, 163.0401, 148.0167, 147.0453, 104.0269, 93.0346, 85.0296 | 121.0295 | C_10_H_7_O_4_ | 191.03498 | 0.19 | 7 | 100 |
| 313.1083 |  | 7.26 | 121.0295 | false positive | 1.14E+07 | 282.1474, 250.1576, 149.0608, 121.0295 | 121.0295 | C_18_H_17_O_5_ | 313.10815 | 0.61 | 10 | 99.79 |
| 271.0613 |  | 7.69 | 121.0295, 165.0193 | ***isomers of mono-2-hydroxy-4-methyl benzoate phthalate (level 3)*** | 3.78E+07 | 165.0193, 137.0245 | 121.0295, 165.0193 | C_15_H_11_O_5_ | 271.0612 | 0.53 | 10 | 99.84 |
| 349.2023 |  | 7.6 | 121.0295, 165.0193 | false positive | 3.98E+06 | 349.2025, 287.2019, 269.0460, 83.0140 | 121.0298 | C_20_H_29_O_5_ | 349.20205 | 0.81 | 6 | 82.4 |
| 263.129 |  | 7.72 | 165.0193 | false positive | 8.67E+05 | 222.9996, 219.1039, 183.0816, 145.0620, 128.0352, 127.0513, 109.0406, 96.9605, 79.9575 | 165.0189 | C_15_H_19_O_4_ | 263.12888 | 0.55 | 6 | 98.66 |
| 265.0719 |  | 7.92 | 121.0295 | false positive | 1.42E+06 | 216.9894, 109.0659, 96.9602 | 121.0292 |  |  |  |  |  |
| 271.0613 |  | 8.04 | 165.0193 | ***isomers of mono-2-hydroxy-4-methyl benzoate phthalate (level 3)*** | 3.68E+07 | 165.0193, 137.0245, 93.0346 | 121.0297, 165.0193 | C_15_H_11_O_5_ | 271.0612 | 0.53 | 10 | 99.84 |
| 269.0457 |  | 8.39 | 121.0295 | false positive | 6.78E+07 | 189.0922, 99.0453, 87.0452, 79.9575, 72.0218, 59.0137, 57.0343 | NIL | C_15_H_9_O_5_ | 269.04555 | 0.51 | 11 | 98.54 |
| 263.129 |  | 8.71 | 121.0295 | false positive | 2.55E+06 | 219.1767, 183.0814, 145.0620, 127.0513, 109.0407, 79.9576 | NIL | C_15_H_19_O_4_ | 263.12888 | 0.55 | 6 | 100 |
| 297.1131 |  | 8.77 | 121.0295 | false positive | 3.47E+08 | 297.1135, 253.1236, 189.0558, 165.0559, 145.0661, 121.0659, 119.0504, 107.0503 | 121.0292 | C_18_H_17_O_4_ | 297.11323 | -0.35 | 10 | 100 |
| 331.192 |  | 8.79 | 121.0295 | false positive | 1.35E+06 | 331.1918, 273.1497, 251.0721m 219.1026, 162.0682, 137.0610, 125.0607, 57.0344 | NIL | C_20_H_27_O_4_ | 331.19148 | 1.7 | 7 | 82.22 |
| 343.1188 |  | 8.76 | 121.0295 | false positive | 5.15E+06 | 263.0463, 167.1078, 113.0245, 85.0296, 72.9931, 71.0139, 59.0137 | 121.0296 | C_19_H_19_O_6_ | 343.11871 | 0.27 | 10 | 97.68 |
| **221.082** |  | **9.49** | **121.0295, 147.0087** | **MiBP^a^ (Level 1)** | **1.56E+06** | **221.0811, 177.0920, 162.9538, 121.0295, 109.0659, 93.0346, 71.0503, 69.0346** | **121.0295, 147.0089** | **C_12_H_13_O_4_** | **221.08193** | **0.24** | **6** | **99.14** |
| 301.0718 |  | 9.67 | 121.0295 | false positive | 3.99E+07 | 301.0725, 221.0454, 165.1281, 151.0037, 134.0374, 131.0135, 107.0139, 79.9575, 74.0248, 71.0502 | 121.0299 | C_16_H_13_O_6_ | 301.07176 | 0.15 | 10 | 100 |
| 407.2078 |  | 9.89 | 121.0295 | false positive | 1.44E+07 | 331.1919, 297.1497, 282.1262, 189.0923, 125.0608 | NIL | C_22_H_31_O_7_ | 407.20753 | 0.58 | 7 | 96.54 |
| 331.1917 |  | 9.92 | 121.0295 | false positive | 4.08E+06 | 331.1920, 297.1496, 282.1264, 221.1544, 189.0923, 149.0608, 125.0608, 107.0499, 97.0658 | NIL | C_20_H_27_O_4_ | 331.19148 | 0.78 | 7 | 97.8 |
| 361.2024 |  | 9.77 | 121.0295 | false positive | 1.09E+06 | 331.1919, 315.1608, 303.1977, 271.1707, 180.9780, 124.0076, 73.0295, 59.0135 | NIL | C_21_H_29_O_5_ | 361.20205 | 0.87 | 7 | 79.2 |
| **221.0819** |  | **9.95** | **121.0295, 147.0089** | **MBP^a^ (Level 1)** | **2.16E+06** | **147.0087, 134.0376, 121.0295, 71.0503, 69.0346** | **121.0295, 147.0087** | **C_12_H_13_O_4_** | **221.08193** | **-0.1** | **6** | **99.14** |
| 327.124 |  | 9.99 | 121.0295, 147.0089 | false positive | 1.45E+06 | 259.1924, 201.1134, 183.0125, 152.0600, 151.0565, 127.1129, 102.0350, 57.0344 | 121.0296 | C_19_H_19_O_5_ | 327.1238 | 0.51 | 10 | 100 |
| **293.1399** |  | **10.38** | **121.0295** | **MEHHP^a^ (Level 1)** | **3.56E+06** | **145.1235, 143.1078, 121.0296** | **121.0296, 147.0089** | **C_16_H_21_O_5_** | **293.13945** | **1.42** | **6** | **100** |
| 357.1346 |  | 10.65 | 121.0295 | false positive | 5.18E+06 | 357.1339, 147.0451, 123.0453, 96.9602, 81.0347 | 121.0297 | C_20_H_21_O_6_ | 357.13436 | 0.62 | 10 | 99.7 |
| 319.1913 |  | 10.87 | 121.0295 | false positive | 1.42E+07 | 319.1917, 275.2019, 85.0297, 71.0140 | NIL | C_19_H_27_O_4_ | 319.19148 | -0.53 | 6 | 100 |
| 293.1397 |  | 11.57 | 121.0295 | false positive | 6.61E+06 | 216.1156, 177.0921, 162.0687, 115.0401, 71.0503 | 121.0294 | C_16_H_21_O_5_ | 293.13945 | 1 | 6 | 99.82 |
| **291.1241** |  | **11.65** | **121.0295** | **MEOHP^a^ (Level 1)** | **2.33E+06** | **143.1078, 121.0295, 113.0972, 99.0816, 71.0502** | **121.0295, 147.0093** | **C_16_H_19_O_5_** | **291.1238** | **1.2** | **7** | **98.34** |
| 307.1553 | 11.92 | 11.73 | 121.0295 | ***mono-3,4 dimethyl-5-ethyl-6-hydroxyhexyl phthalate (level 2)*** | 4.70E+06 | 307.1554, 159.1392, 121.0296 | 121.0296, 147.0091, 165.0208 | C_17_H_23_O_5_ | 307.1551 | 0.67 | 6 | 100 |
| 341.1032 |  | 11.57 | 121.0295 | false positive | 1.34E+07 | 341.1038, 311.0927, 176.0480, 147.0454, 137.0244, 123.0452, 121.0297, 69.0346 | 121.0297 | C_19_H_17_O_6_ | 341.10306 | 0.35 | 11 | 99.73 |
| 265.1082 |  | 12.1 | 121.0295 | false positive | 1.65E+06 | 221.1187, 177.1286, 96.9602 | 121.0296 | C_14_H_17_O_5_ | 265.10815 | 0.37 | 6 | 98.65 |
| 291.1604 |  | 12.16 | 121.0295 | false positive | 7.67E+06 | 291.1605, 247.1705, 148.0531, 135.0453, 122.0374 | 121.0295 | C_17_H_23_O_4_ | 291.16018 | 0.75 | 6 | 99.84 |
| 307.1553 |  | 12.15 | 121.0295 | ***isomers of mono-2-ethyl-6-hydroxyoctyl phthalate (level 3)*** | 2.23E+06 | 227.0899, 211.0584, 121.0296, 96.9602 | 121.0296, 147.0094 | C_17_H_23_O_5_ | 307.1551 | 0.57 | 6 | 98.17 |
| 341.1032 |  | 12.15 | 121.0295 | false positive | 2.94E+06 | 341.1038, 183.0121, 176.0476, 123.0452, 121.0296, 74.0248, 69.0346 | 121.0296 | C_19_H_17_O_6_ | 341.10306 | 0.35 | 11 | 100 |
| 349.2023 |  | 12.15 | 121.0295 | false positive | 1.82E+06 | 349.2024, 331.1917, 303.1969, 269.0457, 96.9602 | NIL | C_20_H_29_O_5_ | 349.20205 | 0.64 | 6 | 0 |
| 407.2077 |  | 12.19 | 121.0295 | ***isomers of dibenzenecarboxylic acid, 3,6-dihydroxy-7-(1,6-dimethyl)-7-cyclohexyl-ester (level 3)*** | 2.53E+06 | 331.1918, 96.9602, 79.9574 | 121.0294, 165.0194 | C_22_H_31_O_7_ | 407.20753 | 0.43 | 7 | 96.99 |
| 351.1817 |  | 12.36 | 121.0295 | false positive | 4.52E+06 | 271.2284, 144.0065, 137.0972, 112.0166, 96.9602, 87.0453, 72.0455, 59.0137, 55.0187 | 121.0297 | C_19_H_27_O_6_ | 351.18131 | 1.02 | 6 | 100 |
| 305.1396 |  | 12.34 | 121.0295 | ***isomers of mono-4-methyl-7-oxo-octyl phthalate (level 3)*** | 2.90E+06 | 145.0296, 121.0296, 119.0502, 71.0503 | 121.0296, 147.0087 | C_17_H_21_O_5_ | 305.13945 | 0.66 | 7 | 98.18 |
| 333.2076 |  | 12.52 | 121.0295, 165.0193 | false positive | 2.79E+06 | 333.2076, 303.1963, 253.0513, 137.0972, 96.9602, 79.9575, 59.0137 | 121.0296 | C_20_H_29_O_4_ | 333.20713 | 1.44 | 6 | 97.79 |
| 349.2019 |  | 12.46 | 121.0295, 165.0193 | false positive | 1.29E+06 | 349.2016, 331.1924, 303.1966, 269.0462, 248.1373, 96.9600, 73.0295, 57.0342 | NIL | C_20_H_29_O_5_ | 349.20205 | -0.41 | 6 | 82.22 |
| 393.2289 |  | 12.49 | 121.0295, 165.0193 | false positive | 1.08E+06 | 313.1643, 298.1406, 297.1336, 96.9602, 79.9575 | 121.0293 | C_22_H_33_O_6_ | 393.22826 | 1.5 | 6 | 100 |
| 321.1708 |  | 12.66 | 121.0295 | ***isomers of mono-6-methyl-7-hydroxy-nonyl phthalate (Level 3)*** | 1.22E+06 | 219.0879, 121.0295, 96.9602, 79.9574 | 121.0295, 147.0090 | C_18_H_25_O_5_ | 321.17075 | 0.19 | 6 | 97.99 |
| 305.1396 | 12.57 | 12.57 | 121.0295 | ***mono-3-propyl-4-oxo-hexyl phthalate (level 2)*** | 1.83E+06 | 121.0296,145.0297, 163.0405,71.0503 | 121.0296, 147.0087 | C_17_H_21_O_5_ | 305.13945 | 0.66 | 7 | 98.18 |
| 307.1189 |  | 12.66 | 121.0295, 165.0193 | ***isomers of mono-1-hydroxy-2-oxo-5ethylhexyl phthalate (Level 3)*** | 3.77E+07 | 233.0820, 219.0663, 218.0585, 215.0714, 204.0428, 187.0764, 165.0193, 137.0244, 123.0089 | 121.0295, 165.0192 | C_16_H_19_O_6_ | 307.11871 | 0.5 | 7 | 99.79 |
| 263.1291 |  | 12.66 | 121.0295, 165.0193 | false positive | 9.43E+06 | 233.0821, 218.0585, 215.0716, 205.0873, 187.0765, 178.0270, 165.0190, 159.0815 | 165.019 | C_15_H_19_O_4_ | 263.12888 | 0.79 | 6 | 100 |
| 289.1078 |  | 12.66 | 121.0295, 165.0193 | false positive | 2.99E+06 | 259.0609, 229.0870, 215.0714, 214.0638, 187.0763 | NIL | C_16_H_17_O_5_ | 289.10815 | -1.03 | 8 | 98.3 |
| 317.1758 |  | 12.69 | 121.0295, 165.0193 | false positive | 5.15E+06 | 317.1765, 299.1641, 273.1863, 255.1758, 229.1601, 163.0767, 145.0300, 135.0453, 123.0453, 122.0373, 93.0346 | 121.0294 | C_19_H_25_O_4_ | 317.17583 | -0.07 | 7 | 100 |
| 305.176 |  | 12.69 | 121.0295, 165.0193 | false positive | 5.23E+06 | 305.1764, 261.1860, 162.0687, 149.0608, 136.0530 | 121.0293 | C_18_H_25_O_4_ | 395.17583 | 0.53 | 6 | 98.15 |
| 349.2023 |  | 12.68 | 121.0295, 165.0193 | false positive | 2.04E+06 | 349.2024, 331.1928, 303.1971, 269.0460, 243.1756, 233.1553, 219.1394, 187.0795, 143.0899, 96.9602, 79.9575, 69.0347 | 121.0299 | C_20_H_29_O_5_ | 349.20205 | 0.64 | 6 | 97.65 |
| 359.1867 |  | 12.66 | 121.0295, 165.0193 | false positive | 1.76E+06 | 359.1884, 315.1973, 287.2014, 285.1862,231.1753, 178.0632, 150.0321, 96.9602, | NIL | C_21_H_27_O_5_ | 359.1864 | 9.78 | 8 | 100 |
| 377.2335 |  | 12.66 | 121.0295, 165.0193 | false positive | 1.28E+06 | 191.0353, 96.9602, 79.9573 | 121.0296 | C_22_H_33_O_5_ | 377.23335 | 0.38 | 6 | 97.29 |
| 393.2286 |  | 12.6 | 121.0295, 165.0193 | false positive | 9.93E+06 | 96.9602, 79.9574 | 121.0296 | C_22_H_33_O_6_ | 393.22826 | 0.8 | 6 | 96.73 |
| 333.2074 |  | 12.98 | 121.0295 | false positive | 8.65E+07 | 333.2076, 144.0064, 137.0973, 119.0866, 112.0166, 71.0503, 69.0346, 55.0187 | NIL | C_20_H_29_O_4_ | 333.20713 | 0.79 | 6 | 100 |
| 255.0665 |  | 12.92 | 121.0295 | false positive | 5.48E+06 | 255.0665, 213.0557, 171.0454, 151.0037, 145.0660, 107.0140, 96.9602, 83.0140, 74.0248, 65.0032 | NIL | C_15_H_11_O_4_ | 255.06628 | 1.03 | 10 | 100 |
| 305.1761 |  | 12.94 | 121.0295 | false positive | 7.42E+06 | 261.1861, 149.0609, 136.0530 | 121.0293 | C_18_H_25_O_4_ | 395.17583 | 0.83 | 6 | 97.95 |
| 311.0922 |  | 12.95 | 121.0295 | false positive | 2.19E+06 | 293.2127, 237.0916, 235.0759, 222.0682, 209.0603, 116.0506 | NIL | C_18_H_15_O_5_ | 311.0925 | -0.97 | 11 | 81.89 |
| 333.2074 |  | 13.1 | 121.0295 | false positive | 1.10E+07 | 333.2076, 144.0064, 137.0973, 119.0866, 112.0166, 71.0503, 69.0346, 55.0187 | NIL | C_20_H_29_O_4_ | 333.20713 | 0.89 | 6 | 99.78 |
| 335.1868 |  | 13.4 | 121.0295 | false positive | 2.10E+07 | 177.0921, 162.0687, 157.0870, 139.0766, 97.0659 | NIL | C_19_H_27_O_5_ | 335.1864 | 1.29 | 6 | 99.76 |
| 285.0771 |  | 13.36 | 121.0295 | false positive | 6.83E+06 | 285.0769, 243.0666, 196.0016, 164.0116, 151.0037, 136.0166, 108.0218, 107.0139, 83.0139 | NIL | C_16_H_13_O_5_ | 285.07685 | 0.95 | 10 | 98.37 |
| 319.1917 |  | 13.43 | 121.0295 | false positive | 8.01E+06 | 319.1913, 275.2017, 163.0766, 150.0687, 149.0609 | 121.0294 | C_19_H_27_O_4_ | 319.19148 | 0.53 | 6 | 99.8 |
| 461.2913 |  | 14.45 | 121.0295 | false positive | 3.14E+06 | 461.2915 | NIL | C_27_H_41_O_6_ | 461.29086 | 0.86 | 7 | 100 |
| 357.171 |  | 14.5 | 121.0295 | false positive | 2.60E+06 | 357.1711, 339.1604, 313.1814, 311.1652, 259.1339, 241.1237, 205.0869, 95.0503, 71.0503 | 121.0296 | C_21_H_25_O_5_ | 357.17075 | 0.77 | 9 | 97.48 |
| **277.1448** |  | **14.43** | **121.0295** | **MEHP^a^ (Level 1)** | **2.56E+06** | **147.0089, 134.0375, 127.1129, 121.0296, 93.0345, 79.9574** | **121.0296, 147.0089** | **C_16_H_21_O_4_** | **277.14453** | **0.88** | **6** | **98.5** |
| 459.2732 |  | 14.63 | 121.0295 | false positive | 1.61E+06 | 391.2859, 345.2803, 343.2647, 96.9602, 79.9574 | NIL | C_27_H_39_O_6_ | 459.27521 | -4.33 | 8 | 95.6 |
| 385.2386 |  | 15.36 | 121.0295 | false positive | 8.71E+06 | 385.2388, 367.2280, 341.2487, 323.2381, 317.2129, 283.2070, 215.1441, 123.0817, 57.0343 | NIL | C_24_H_33_O_4_ | 385.23843 | 0.48 | 8 | 100 |
| 291.1606 | 15.58 | 15.51 | 121.0295, 147.0089 | ***mono-7-methyloctyl phthalate (level 2)*** | 8.92E+05 | 147.0088, 141.1286, 134.0373, 121.0295, 79.9574 | 121.0295, 147.0088, 134.0373 | C_17_H_23_O_4_ | 291.16018 | 1.48 | 6 | 98.33 |
| 349.1657 |  | 15.86 | 165.0193 | false positive | 2.77E+07 | 275.1292, 220.0743, 180.0429, 167.0350, 166.0271 | 165.0194 | C_19_H_25_O_6_ | 349.16566 | 0.23 | 7 | 100 |
| 329.176 |  | 15.9 | 165.0193 | false positive | 3.36E+06 | 329.1761, 285.1863, 257.1913, 255.1756, 185.1336, 131.0866, 123.0817 | NIL | C_20_H_25_O_4_ | 329.17583 | 0.49 | 8 | 99.78 |
| 305.1761 |  | 15.85 | 165.0193 | false positive | 8.81E+05 | 289.1663, 275.1299, 219.0878, 203.0567, 180.0428, 167.0350, 166.0271, 79.9574 | 165.0193 | C_18_H_25_O_4_ | 305.17583 | 0.93 | 6 | 100 |
| 375.1815 |  | 18.65 | 165.0193 | false positive | 1.16E+07 | 301.1449, 233.0818, 219.0664, 218.0585, 204.0426, 201.0558, 167.0351, 151.0400, 137.0246 | 165.0194 | C_21_H_27_O_6_ | 375.18131 | 0.39 | 8 | 100 |
| *197.064* |  | 6.75 | *124.0396* | *MEP (ring-1,2-^13^C_2_, dicarboxyl-^13^C_2_)* | 1.13E+06 | 162.8392, 160.8422, 137.0244, 124.0396 | 124.0396, 151.0222 | C_6_^13^C_4_H_9_O_4_ | 197.0637 | 1.52 | 6 | 0 |
| *225.0954* |  | 9.96 | *124.0396, 151.0220* | *MBP (ring-1,2-^13^C_2_, dicarboxyl-^13^C_2_)* | 4.73E+06 | 151.0221, 137.0474, 124.0396, 72.0536, 71.0503, 70.0379, 69.0346 | 124.0396, 151.0221 | ^13^C_4_C_8_H_13_O_4_ | 225.09535 | 0 | 6 | 99.13 |
| *297.153* |  | 10.39 | *124.0396, 151.0220, 169.0328* | *MEHHP (ring-1,2-^13^C_2_, dicarboxyl-^13^C_2_)* | 1.44E+07 | 124.0396 | 124.0396, 151.0224 | ^13^C_4_C_12_H_21_O_5_ | 297.15287 | 0.4 | 6 | 98.14 |
| *311.1326* |  | 10.85 | *124.0396, 151.0220, 169.0328* | *MCMHP (ring-1,2-^13^C_2_, dicarboxyl-^13^C_2_)* | 9.58E+05 | 311.1331, 293.2123, 237.1860, 211.1340, 183.0123, 159.1028, 124.0396, 113.0973, 59.0137 | 124.0396, 169.0335 | C_12_^13^C_4_H_19_O_6_ | 311.13213 | 0.62 | 7 | 85.25 |
| *295.1372* |  | 11.68 | *124.0396, 151.0220, 169.0328* | *MEOHP (ring-1,2-^13^C_2_, dicarboxyl-^13^C_2_)* | 1.50E+07 | 143.1078, 124.0396, 113.0973, 99.0817, 71.0503 | 124.0396, 151.0220 | ^13^C_4_C_12_H_19_O_5_ | 295.13722 | -0.03 | 7 | 98.15 |
| *251.1111* |  | 12.18 | *124.0396, 151.0220, 169.0328* | *MCyHP (ring-1,2-^13^C_2_, dicarboyxl-^13^C_2_)* | 2.90E+07 | 151.0223, 124.0396, 97.0660, 95.0503 | 124.0396, 151.0223 | ^13^C_4_C_10_H_15_O_4_ | 251.111 | 0.51 | 7 | 0 |
| *281.1579* |  | 14.43 | *124.0396, 151.0220* | *MEHP (ring-1,2-^13^C_2_, dicarboxyl-^13^C_2_)* | 5.00E+07 | 151.0220, 137.0474, 127.1130, 124.0395, 95.0413, 77.0306 | 124.0395, 151.0220 | ^13^C_4_C_12_H_21_O_4_ | 281.15795 | -0.19 | 6 | 98.34 |
| *295.1734* |  | 14.95 | *124.0396, 151.0220, 169.0328* | *7-MMOP (ring-1,2-^13^C_2_, dicarboxyl-^13^C_2_)* | 5.29E+07 | 151.0223, 142.1320, 141.1286, 139.1131, 137.0476, 124.0396, 69.0345 | 124.0396, 151.0223, 169.0325 | ^13^C_4_C_13_H_23_O_4_ | 295.1736 | -0.58 | 6 | 0 |
| *311.1688* |  | 14.67 | *124.0396, 151.0220, 169.0328* | non-spiked | 2.98E+07 | 311.1689, 184.0200, 183.0121 | NIL | ^13^C_4_C_13_H_23_O_5_ | 311.16852 | 0.79 | 6 | 0 |
| *311.1687* |  | 14.95 | *124.0396, 151.0220, 169.0328* | non-spiked | 2.12E+06 | 311.1689, 197.0279, 184.0199, 183.0121 | NIL | ^13^C_4_C_13_H_23_O_5_ | 311.16852 | 0.69 | 6 | 0 |
| *281.1578* |  | 15.09 | *124.0396, 151.0220, 169.0328* | *(MOP) (ring-1,2-^13^C_2_, dicarboxyl-^13^C_2_)* | 5.54E+07 | 151.0223, 137.0473, 128.1162, 127.1129, 124.0396, 77.0307 | 124.0396, 151.0223, 169.0334 | ^13^C_4_C_12_H_21_O_4_ | 281.15795 | -0.52 | 6 | 1.52 |
| *309.1892* |  | 17.05 | *124.0396, 151.0220, 169.0328* | *3,7-diMMOP (ring-1,2-^13^C_2_, dicarboxyl-^13^C_2_)* | 1.29E+08 | 156.1472, 155.1443, 154.1322, 153.1285, 151.0223, 139.1131, 137.0474, 124.0395, 69.0345 | 124.0395, 151.0223, 169.0334 | ^13^C_4_C_14_H_25_O_4_ | 309.18925 | -0.24 | 6 | 0 |

Peak filter was set up as relative height of no less than 10 % of largest peak

*Italicized names and red color*: isotopically labelled compound

^a^: Confirmed with authentic standards (highlighted with orange )

^b^:Not confirmed with standards (highlighted with green)

Highlighted in yellow color are non-spiked labelled compounds

Table S5, List of precursor ions identified in pooled urine (infertile men) with the DDA data

| m/z | Predicted RT (min) | RT (min) | EIC ions | Identified compounds (Identification level) | Peak intensity (counts) | Major ions | observed diagnostic ions | calculated formula | Theo Mass | Difference (ppm) | RDB | Pattern Cov. (%) |
| --- | --- | --- | --- | --- | --- | --- | --- | --- | --- | --- | --- | --- |
| 193.0507 |  | 5.94 | 121.0295, 165.0193 | ***isomers of 1-ethyl-phthalic acid (level 3)*** | 2.13E+06 | 193.0509, 178.0275, 163.0404, 149.0609, 137.0246, 134.0376, 123.0453, 121.0298, 93.0347 | 121.0298, 165.0199 | C_10_H_9_O_4_ | 193.05063 | 0.38 | 6 | 100 |
| 293.1036 |  | 6.27 | 121.0295 | false positive | 3.93E+06 | 293.1036, 261.0775, 213.0923, 190.0274, 189.0196, 177.1287 | 121.0298 | C_15_H_17_O_6_ | 293.10306 | 1.86 | 7 | 98.32 |
| 193.0508 |  | 6.29 | 121.0295 | false positive | 1.18E+06 | 193.0509, 178.0638, 161.0244, 149.0609, 139.0402, 137.0609, 135.0454, 134.0375, 133.0293, 121.0298, 93.0347 | 121.0299 | C_10_H_9_O_4_ | 193.05063 | 0.77 | 6 | 90.24 |
| 237.0771 |  | 6.33 | 121.0295 | false positive | 1.82E+06 | 193.0874, 165.0922, 164.0845, 108.0219, 96.9602, 79.9575 | 121.0297 | C_12_H_13_O_5_ | 237.07685 | 0.95 | 6 | 0 |
| 313.1086 |  | 6.44 | 121.0295 | false positive | 4.67E+06 | 147.0454, 123.0454, 121.0660, 119.0503, 81.0347 | 121.0298 | C_18_H_17_O_5_ | 313.10815 | 1.58 | 10 | 98.02 |
| 301.1449 |  | 6.47 | 121.0295 | false positive | 2.90E+07 | 301.1451, 271.1345, 253.1238, 241.1239, 121.0297, 107.0504 | 121.0297, | C_18_H_21_O_4_ | 301.14453 | 1.21 | 8 | 100 |
| 305.1399 |  | 6.51 | 121.0295 | false positive | 5.10E+05 | 151.0766, 135.7998, 121.0296, 109.0659, 69.8867, 59.0131, 57.0344 | 121.0296 | C_17_H_21_O_5_ | 305.13945 | 1.36 | 7 | 84.47 |
| 285.0405 |  | 6.65 | 147.0089 | false positive | 4.23E+06 | 285.0413, 217.0508, 205.0872, 199.0404, 175.0403, 161.0973, 133.0296, 83.0140, 65.0033 | 147.0093 | C_15_H_9_O_6_ | 285.04046 | 0.12 | 11 | 98.25 |
| 313.1087 |  | 6.66 | 121.0295 | false positive | 1.07E+07 | 313.1088, 269.1186, 227.1082, 205.0509, 161.0611, 149.0611, 137.0610, 131.0504, 119.0503, 107.0504, 95.0504 | 121.0297 | C_18_H_17_O_5_ | 313.10815 | 1.78 | 10 | 99.79 |
| 191.0352 |  | 6.75 | 147.0089 | false positive | 2.33E+06 | 191.0352, 176.0117, 148.0168 | 147.0091 | C_10_H_7_O_4_ | 191.03498 | 1.07 | 7 | 99.24 |
| **193.0507** |  | **6.77** | **121.0295, 147.0089** | **MEP^a^ (Level 1)** | **3.47E+06** | **193.0509, 175.0767, 165.0016, 149.0610, 137.0246, 134.0376, 123.0452, 121.0296, 119.0502, 107.0502, 93.0347, 79.9574** | **121.0296, 147.0088** | **C_10_H_9_O_4_** | **193.05063** | **0.38** | **6** | **99.24** |
| 255.0666 |  | 6.81 | 121.0295 | false positive | 9.30E+06 | 255.0666, 149.0246, 135.0090, 121.0297, 91.0191 | 121.0297 | C_15_H_11_O_4_ | 255.06628 | 1.38 | 10 | 98.71 |
| 313.1087 |  | 6.85 | 121.0295 | false positive | 1.03E+07 | 313.1089, 269.1185, 161.0611, 147.0818, 121.0297, 109.0296 | 121.0297 | C_18_H_17_O_5_ | 313.10815 | 1.68 | 10 | 100 |
| 193.0507 |  | 6.97 | 121.0295 | false positive | 6.83E+05 | 193.0508, 175.0402, 149.0609, 131.0504, 107.0504, 105.0711 | 121.0295 | C_10_H_9_O_4_ | 193.05063 | 0.22 | 6 | 90.24 |
| 313.1086 |  | 7.26 | 121.0295 | false positive | 7.13E+06 | 313.1101, 121.0296, 107.0504, 93.0347, 83.0140 | 121.0296 | C_18_H_17_O_5_ | 313.10815 | 1.58 | 10 | 100 |
| 191.0352 |  | 7.46 | 147.0089 | false positive | 1.87E+06 | 191.0353, 176.0117, 148.0168 | 147.0092 | C_10_H_7_O_4_ | 191.03498 | 0.99 | 7 | 90.24 |
| 285.1136 |  | 7.53 | 121.0295 | false positive | 2.66E+06 | 267.1033, 241.1239, 192.0430, 160.0533, 149.0601, 147.0457, 121.0297 | 121.0297 | C_17_H_17_O_4_ | 285.11323 | 1.24 | 9 | 98.34 |
| 271.0617 |  | 7.68 | 121.0295, 165.0193 | ***isomers of hydroxybenzyl phthalate (level 3)*** | 2.98E+06 | 191.0353, 165.0195, 137.0244 | 121.0295, 165.0195 | C_15_H_11_O_5_ | 271.0612 | 1.77 | 10 | 100 |
| 309.1348 |  | 7.98 | 165.0193 | false positive | 2.49E+06 | 235.0979, 299.0867, 180.0431, 167.0351, 166.0272, 163.0406 | 165.0191 | C_16_H_21_O_6_ | 309.13436 | 1.31 | 6 | 83.46 |
| 271.0616 |  | 8.03 | 121.0295, 165.0193 | ***isomers of mono-2-hydroxy-4-methyl benzoate phthalate (level 3)*** | 6.28E+07 | 271.0617, 177.0195, 151.0039, 151.0039, 119.0504, 107.0140, 93.0347, 83.0140, 65.0033 | 121.0294, 165.0198 | C_15_H_11_O_5_ | 271.0612 | 1.43 | 10 | 99.84 |
| 269.0459 |  | 8.38 | 121.0295 | false positive | 1.53E+07 | 269.0461, 177.1287, 133.0297 | 121.0295 | C_15_H_9_O_5_ | 269.04555 | 1.19 | 11 | 99.84 |
| 297.1134 |  | 8.76 | 121.0295 | false positive | 4.08E+08 | 297.1138, 189.0560, 145.0661, 133.0662, 121.0660, 119.0504, 107.0504 | 121.0296 | C_18_H_17_O_4_ | 297.11323 | 0.68 | 10 | 100 |
| 301.0721 |  | 8.94 | 165.0193 | false positive | 2.73E+06 | 301.0716, 221.0461, 196.0019, 164.0117, 151.0040, 149.0611, 136.0169, 134.0376, 108.0219, 107.0140, 83.0139, 65.0032, 63.0239 | 165.0198 | C_16_H_13_O_6_ | 301.07176 | 1.27 | 10 | 98.18 |
| 327.1243 |  | 9.2 | 121.0295 | false positive | 3.99E+07 | 327.1250, 283.1361, 189.0561, 183.0125, 161.0609, 147.0455, 137.0609, 135.0453, 133.0660, 123.0454, 122.0375, 121.0298, 119.0502, 107.0505, 93.0347, 83.0140 | 121.0298 | C_19_H_19_O_5_ | 327.1238 | 1.53 | 10 | 97.85 |
| 297.1136 |  | 9.44 | 121.0295 | false positive | 1.48E+06 | 297.1142, 253.1246. 189.0560, 145.0653, 133.0663, 121.0664, 119.0504, 107.0505 | 121.0296 | C_18_H_17_O_4_ | 297.11323 | 1.09 | 10 | 82.03 |
| 221.0822 |  | 9.49 | 121.0295, 147.0089 | false positive | 1.06E+06 | 177.0927, 147.0085, 141.0194, 134.0375, 123.0091, 121.0299, 113.0246, 107.0504, 99.3997, 95.0140, 79.9574, 71.0502 | 121.0299, 147.0085 | C12H13O4 | 221.08193 | 1.28 | 6 | 100 |
| 301.0721 |  | 9.67 | 165.0193 | false positive | 9.61E+07 | 301.0724, 285.0406, 242.0589, 201.0195, 196.0015, 174.0324, 164.0118, 151.0040, 136.0168, 134.0377, 108.0219, 107.0140, 83.0140, 65.0033 | 165.0193 | C_16_H_13_O_6_ | 301.07176 | 1.07 | 10 | 99.79 |
| **221.0822** |  | **9.97** | **121.0295, 147.0089** | **MBP^a^ (Level 1)** | **1.21E+06** | **147.0090, 141.0194, 134.0376, 123.0089, 121.0294, 113.0245, 95.0138, 71.0503, 69.0347** | **121.0294, 147,0090** | **C_12_H_13_O_4_** | **221.08193** | **1.07** | **6** | **100** |
| 327.1243 |  | 10 | 121.0295 | false positive | 1.71E+06 | 327.1257, 312.1026, 268.1114, 267.1032, 189.0561, 183.0121, 161.0610, 147.0455, 137.0611, 122,0377, 121.0297, 119.0504, 107.0503, 96.9602 | 121.0297 | C_19_H_19_O_5_ | 327.1238 | 1.63 | 10 | 97.85 |
| 421.1875 |  | 10.36 | 121.0295 | false positive | 1.63E+06 | 390.0447, 375.1816, 345.1711, 341.1031, 327.1601, 311.0938, 293.0826, 265.0880, 176.0481, 161.0610, 123.0454, 121.0292, 79.9573, 69.0346 | 121.0292 | C_22_H_29_O_8_ | 421.18679 | 1.68 | 8 | 100 |
| 293.14 |  | 10.39 | 121.0295 | false positive | 3.17E+06 | 221.1554, 143.1078, 121.0297, 99.0816 | 121.0297 | C_16_H_21_O_5_ | 293.13945 | 2.04 | 6 | 98.34 |
| 307.1556 |  | 10.6 | 121.0295 | false positive | 2.23E+06 | 171.1030, 153.0925, 121.0297, 111.0817, 108.0219 | 121.0297 | C_17_H_23_O_5_ | 305.1551 | 1.57 | 6 | 98.17 |
| 357.1349 |  | 10.64 | 121.0295 | false positive | 2.40E+06 | 168.0428, 136.0531, 122.0375, 121.0297, 83.0140 | 121.0297 | C_20_H_21_O_6_ | 357.13436 | 1.64 | 10 | 97.51 |
| 293.14 |  | 10.71 | 121.0295 | false positive | 6.50E+05 | 266.9727, 206.2309, 193.6036, 145.1233, 144.5153, 143.1080, 129.7545, 121.0295, 102.9276, 101.3132, 85.0660, 78.0450 | 121.0295 | C_16_H_21_O_5_ | 293.13945 | 1.73 | 6 | 85.25 |
| *293.14* |  | *11.57* | *121.0295* | ***isomers of MEHHP^b^ (Level 3)*** | *6.10E+06* | *231.1390, 216.1158, 177.0923, 162.0688, 115.0404, 71.0503* | *121.0298, 165.0912* | *C_16_H_21_O_5_* | *293.13945* | *1.73* | *6* | *100* |
| 341.1035 |  | 11.59 | 121.0295 | false positive | 2.02E+07 | 341.1041, 176.0481, 175.0405, 147.0452, 123.0453, 122.0375, 121.0297, 109.0295, 69.0347 | 121.0297 | C_19_H_17_O_6_ | 341.10306 | 1.15 | 11 | 100 |
| 291.1242 |  | 11.64 | 121.0295 | false positive | 2.20E+06 | 143.1082, 121.0296, 113.0817, 99.0817, 71.0503 | 121.0296 | C_16_H_19_O_5_ | 291.1238 | 1.41 | 7 | 98.34 |
| 307.1555 | 11.96 | 11.7 | 121.0295, 165.0193 | ***mono-3,4-dimethyl-5-ethyl-6-hydroxyhexyl phthalate (Level 2)*** | 1.92E+06 | 121.0297, 96.9604 | 121.0297, 165.0202 | C_17_H_23_O_5_ | 307.1551 | 1.47 | 6 | 98.17 |
| 307.1553 |  | 12.05 | 121.0295 | false positive | 1.16E+06 | 157.9662, 128.6744, 123.8614, 121.0297, 113.0975, 108.6691, 106.3786, 71.0502, 83.6031, 66.1355 | 121.0297 | C_17_H_23_O_5_ | 307.1551 | 0.77 | 6 | 84.47 |
| 341.1035 |  | 12.21 | 121.0295 | false positive | 4.37E+06 | 341.1042, 263.0717, 183.0123, 176.0481, 175.0402, 159.0456, 147.0452, 137.0246, 123.0453, 122.0376, 121.0296, 109.0298, 93.0347, 83.0139, 69.0347 | 121.0296 | C_19_H_17_O_6_ | 341.10306 | 1.15 | 11 | 97.69 |
| 321.1711 |  | 12.21 | 121.0295 | false positive | 9.38E+05 | 173.1185, 163.0764, 148.0529, 127.1130, 121.0296, 109.7957, 97.0660, 95.0504, 71.0501, 57.0344 | 121.0296 | C_18_H_25_O_5_ | 321.17075 | 1.14 | 6 | 100 |
| 305.1399 |  | 12.32 | 121.0295 | false positive | 1.20E+06 | 145.0296, 121.0297, | 121.0297 | C_17_H_21_O_5_ | 305.13945 | 1.46 | 7 | 84.47 |
| 351.1819 |  | 12.35 | 121.0295, 165.0193 | ***isomers of mono 1-propyl-2,4-dimethyloctyl phthalate (level 3)*** | 4.69E+06 | 260.1424, 167.0352, 152.0481, 151.0403, 137.0248, 125.0248, 96.9604 | 121.0297, 165.0197 | C_19_H_27_O_6_ | 351.18131 | 1.72 | 6 | 97.66 |
| 263.1294 |  | 12.47 | 165.0193 | false positive | 5.47E+05 | 195.0667, 180.0430, 165.0195, 137.0246 | 165.0195 | C_15_H_19_O_4_ | 263.12888 | 1.83 | 6 | 86.04 |
| 321.171 |  | 12.48 | 121.0295, 147.0089 | ***isomer of mono-6-methyl-7-hydroxy-nonyl phthalate (Level 3)*** | 9.37E+05 | 243.2374, 147.0088, 121.0297, 118.8868, 112.7870, 105.0970, 84.9448, 73.3710, 57.0344 | 121.0297, 147.0088 | C_18_H_25_O_5_ | 321.17075 | 0.76 | 6 | 83.71 |
| 341.1034 |  | 12.48 | 121.0295 | false positive | 2.05E+06 | 341.1042, 199.0072, 189.0563, 176.0482, 175.0402, 147.0455, 137.0244, 135.0450, 123.0455, 122.0375, 121.0296, 109.0298, 69.0347 | 121.0296 | C_19_H_17_O_6_ | 341.10306 | 1.06 | 11 | 81.02 |
| 307.1192 |  | 12.65 | 121.0295, 165.0193 | ***isomers of mono-1-hydroxy-2-oxo-5ethylhexyl phthalate (Level 3)*** | 3.32E+07 | 233.0822, 219.0665, 218.0587, 215.0716, 204.0432, 201.0563, 187.0767, 165.0195, | 121.0299, 165.0195 | C_16_H_19_O_6_ | 307.11871 | 1.5 | 7 | 99.79 |
| 305.1764 |  | 12.7 | 121.0295 | false positive | 3.92E+06 | 305.1767, 261.1861, 162.0690, 149.0610, 135.0531 | 121.0297 | C_18_H_25_O_4_ | 305.17583 | 1.73 | 6 | 98.16 |
| 391.1771 |  | 12.81 | 165.0193 | false positive | 2.20E+06 | 299.1297, 249.0778, 234.0542, 217.0508, 216.0432, 204.0425, 189.0558, 178.0272, 167.0353, 165.1283, 152.0115, 137.0236, 125.0246, 96.9603, 83.0503 | 165.0199 | C_21_H_27_O_7_ | 391.17623 | 2.22 | 8 | 100 |
| 341.1035 |  | 12.91 | 121.0295 | false positive | 2.63E+06 | 341.1039, 176.0481, 175.0398, 154.0275, 147.0454, 137.0246, 123.0454, 121.0298, 109.0295, 74.0249, 69.0347 | 121.0298 | C_19_H_17_O_6_ | 341.10306 | 1.33 | 11 | 100 |
| 377.1975 |  | 13.47 | 121.0295, 165.0193 | ***isomers of mono-3-butyl-9-carboxynonyl phthalate (level 3)*** | 1.06E+07 | 303.1611, 220.0743, 203.0718, 189.0557, 167.0352, 152,0116, 135.0454 | 121.0293, 165.0195 | C_21_H_29_O_6_ | 377.19696 | 1.45 | 7 | 99.68 |
| **277.145** |  | **14.42** | **121.0295, 147.0089** | **MEHP^a^ (Level 1)** | **2.60E+06** | **147.0090, 135.0455, 134.0375, 127.1131, 121.0298, 93.0346, 75.0241** | **121.0298, 147.0090** | **C_16_H_21_O_4_** | **277.14453** | **1.54** | **6** | **98.5** |
| 291.1607 |  | 15.49 | 121.0295 | false positive | 3.86E+05 | 192.3717, 154.3379, 146.4283, 141.1290, 121.0298, 98.2385, 97.7781, 71.5927 | 121.0928 | C_17_H_23_O_4_ | 291.16018 | 1.69 | 6 | 98.33 |
| 349.1662 |  | 15.85 | 165.0193 | false positive | 2.48E+07 | 275.1295, 220.0745, 180.0431, 167.0352, 166.0275 | 165.0196 | C_19_H_25_O_6_ | 349.16566 | 1.19 | 7 | 100 |
| 375.1817 |  | 18.65 | 165.0193 | false positive | 8.30E+06 | 301.1450, 233.0822, 219.0666, 218.0587, 204.0430, 203.0351, 201.0559, 167.0352, 166.0271, 165.0195, 137.0246 | 165.0195 | C_21_H_27_O_6_ | 375.18131 | 1.04 | 8 | 100 |
| 197.0642 |  | 6.76 | 124.0396, 151.0220 | *MMP (ring-1,2-^13^C_2_, dicarboxyl-^13^C_2_)* | 1.50E+06 | 162.8394, 160.8422, 153.0922, 151.0226, 137.0246, 124.0396, 121.0571, 95.0413 | 124.0396, 151.0228 | C_6_^13^C_4_H_9_O_4_ | 197.0637 | 2.53 | 6 | 0 |
| 225.0957 |  | 9.95 | 124.0396, 151.0220 | *MBP (ring-1,2-^13^C_2_, dicarboxyl-^13^C_2_)* | 4.00E+06 | 151.0224, 137.0478, 124.0398, 96.9600, 72.0537, 71.0503, 70.0379, 69.0347 | 124.0398, 151.0224 | C_8_^13^C_4_H_13_O_4_ | 225.09535 | 1.36 | 6 | 99.13 |
| 297.1532 |  | 10.39 | 124.0396, 151.0220 | MEHHP (ring-1,2-^13^C_2_, dicarboxyl-^13^C_2_) | 1.30E+07 | 143.1077, 124.0397, 107.0503, 99.0816 | 124.0397, 151.0225 | C_12_^13^C_4_H_21_O_5_ | 297.15287 | 1.22 | 6 | 98.14 |
| 311.1327 |  | 10.85 | 124.0396 | MECPP (ring-1,2-^13^C_2_, dicarboxyl-^13^C_2_) | 1.20E+06 | 237.1875, 159.1029, 124.0397, 113.0974 | 124.0396 | C_12_^13^C_4_H_19_O_6_ | 311.13213 | 1.11 | 7 | 0 |
| 295.1375 |  | 11.64 | 124.0396, 151.0220 | MECPP (ring-1,2-^13^C_2_, dicarboxyl-^13^C_2_) | 1.70E+07 | 143.1078, 124.0397, 113.0974, 99.0815, 71.0503 | 124.0397, 151.0222 | C_12_^13^C_4_H_19_O_5_ | 295.13722 | 0.9 | 7 | 98.15 |
| 259.0799 |  | 11.91 | 124.0396, 151.0220 | *MBzP (ring-1,2-^13^C_2_, dicarboxyl-^13^C_2_)* | 2.20E+06 | 237.6200, 151.0228, 141.1288, 136.1006, 134.5002, 124.0401, 123.0367, 121.0292, 107.0504, 101.0607, 91.9249, 77.0309, 71.0502, 51.9974 | 124.0401, 151.0228 | C_11_^13^C_4_H_11_O_4_ | 259.0797 | 0.39 | 10 | 0 |
| 251.1113 |  | 12.2 | 124.0396, 151.0220 | *MCyHP (ring-1,2-^13^C_2_, dicarboyxl-^13^C_2_)* | 2.80E+07 | 151.0224, 124.0397, 97.0660, 95.0504 | 124.0397, 151.0224 | C_10_^13^C_4_H_15_O_4_ | 251.111 | 1.3 | 7 | 0 |
| 339.1639 |  | 14.01 | 124.0396, 151.0220 | non-spiked | 8.00E+05 | 141.0920, 124.0398, 123.0818, 84.0217 | 124.0398, 151.0228 | C_14_^13^C_4_H_23_O_6_ | 339.16343 | 1.5 | 7 | 0 |
| 281.1582 |  | 14.43 | 124.0396, 151.0220, 169.0328 | *MEHP (ring-1,2-^13^C_2_, dicarboxyl-^13^C_2_)* | 5.50E+07 | 151.0227, 137.0477, 128.1164, 127.1131, 124.0397, 95.0413, 77.0307 | 124.0397, 151.0227 | C_12_^13^C_4_H_21_O_4_ | 281.15795 | 1.11 | 6 | 98.34 |
| 295.1739 |  | 14.95 | 124.0396, 151.0220, 169.0328 | *7-MMOP (ring-1,2-^13^C_2_, dicarboxyl-^13^C_2_)* | 5.00E+07 | 151.0226, 142.1319, 141.1289, 140.1164, 139.1131, 137.0477, 124.0398, 69.0347 | 151.0226, 124.0398 | C_13_^13^C_4_H_23_O_4_ | 295.1736 | 0.87 | 6 | 0 |
| 281.1582 |  | 15.08 | 124.0396, 151.0220 | *(MOP) (ring-1,2-^13^C_2_, dicarboxyl-^13^C_2_)* | 5.50E+07 | 151.0223, 137.0477, 128.1163, 127.1131, 125.0974, 124.0398, 95.0414, 77.0310 | 124.0398, 151.0223 | C_12_^13^C_4_H_21_O_4_ | 281.15795 | 0.68 | 6 | 0 |
| 309.1895 |  | 17.06 | 124.0396, 151.0220 | *3,7-diMMOP (ring-1,2-^13^C_2_, dicarboxyl-^13^C_2_)* | 1.30E+08 | 156.1476, 155.1443, 154.1319, 153.1283, 151.0225, 137.0474, 124.0396, 77.0308 | 124.0396, 151,0225 | C_14_^13^C_4_H_25_O_4_ | 309.18925 | 0.75 | 6 | 0 |

Peak filter was set up as relative height of no less than 10 % of largest peak

*Italicized names and red color*: isotopically labelled compound

^a^: Confirmed with authentic standards (highlighted with orange)

^b^:Not confirmed with standards (highlighted with green)

Highlighted in yellow color are non-spiked labelled compounds

Table S6, List of precursor ions identified in pooled urine (fertile men) with the DDA data

| m/z | Predicted RT (min) | RT (min) | EIC ions | Identified compounds (Identification level) | Peak intensity (counts) | Major ions | observed diagnostic ions | Calculated formula | Theo Mass (m/z) | Difference (ppm) | RDB | Pattern Cov. (%) |
| --- | --- | --- | --- | --- | --- | --- | --- | --- | --- | --- | --- | --- |
| 301.1448 |  | 6.46 | 121.0295 | false positive | 7.50E+07 | 301.1448, 271.1343, 253.1237, 241.1238, 121.0297, 107.0503 | 121.0297 | C_18_H_21_O_4_ | 301.14453 | 0.81 | 8 | 99.82 |
| 193.0506 |  | 6.5 | 121.0295, 165.0193 | ***isomers of 1-ethyl-phthalic acid (level 3)*** | 4.08E+06 | 193.0509, 149.0609, 134.0375, 133.0297, 121.0295, 93.0347 | 121.0295, 165.0190 | C_10_H_9_O_4_ | 193.05063 | 0.06 | 6 | 100 |
| 313.1086 |  | 6.66 | 121.0295 | false positive | 1.10E+07 | 313.1088, 269.1187, 205.0509, 161.0609, 137.0609, 107.0504, 95.0504 | 121.0299 | C_18_H_17_O_5_ | 313.10815 | 1.49 | 10 | 98 |
| **193.0507** |  | **6.77** | **121.0295, 147.0089** | **MEP^a^ (Level 1)** | **2.94E+06** | **193.0507, 175.0399, 161.0246, 149.0609, 137.0376, 134.0376, 123.0502, 121.0296, 93.0346** | **121.0296, 147.0085** | **C_10_H_9_O_4_** | **193.05063** | **0.14** | **6** | **100** |
| 255.0665 |  | 6.81 | 121.0295 | false positive | 5.96E+07 | 255.0667, 149.0245, 135.0089, 121.0296, 91.0190 | 121.0296 | C_15_H_11_O_4_ | 255.06628 | 0.97 | 10 | 98.56 |
| 313.1085 |  | 6.84 | 121.0295 | false positive | 9.84E+06 | 313.1088, 269.1188, 161.0612, 147.0817, 121.0296, 109.0296 | 121.0296 | C_18_H_17_O_5_ | 313.10815 | 1.19 | 10 | 98.03 |
| 193.0507 |  | 6.98 | 121.0295 | false positive | 2.29E+07 | 193.0508, 175.0401, 149.0609, 131.0504, 107.0504, 105.0710 | 121.0295 | C_10_H_9_O_4_ | 193.05063 | 0.14 | 6 | 100 |
| 313.1085 |  | 7.26 | 121.0295 | false positive | 7.85E+06 | 313.1090, 191.0716, 121.0296, 107.0504, 93.0347, 83.0140 | 121.0296 | C_18_H_17_O_5_ | 313.10815 | 1.19 | 10 | 98.03 |
| 271.0615 |  | 7.67 | 121.0295, 165.0193 | ***isomers of mono-2-hydroxy-4-methyl benzoate phthalate (level 3)*** | 4.09E+07 | 165.0194, 137.0245 | 121.0295, 165.0194 | C_15_H_11_O_5_ | 271.0612 | 1.21 | 10 | 100 |
| 207.0664 |  | 7.85 | 121.0295 | false positive | 7.90E+05 | 207.0672, 163.0766, 125.0244, 122.0374 | 121.03 | C_11_H_11_O_4_ | 207.06628 | 0.75 | 6 | 100 |
| 283.0615 |  | 7.92 | 147.0089 | false positive | 4.52E+06 | 268.0381, 240.0432, 196.0532 | 147.0089 | C_16_H_11_O_5_ | 283.0612 | 1.05 | 11 | 100 |
| 271.0615 |  | 8.05 | 121.0295, 165.0193 | ***isomers of mono-2-hydroxy-4-methyl benzoate phthalate (level 3)*** | 3.79E+07 | 151.0038, 119.0503, 107.0140, 93.0346, 83.0140, 65.0033 | 121.0293, 165.0198 | C_15_H_11_O_5_ | 271.0612 | 1.21 | 10 | 99.84 |
| 297.1133 |  | 8.76 | 121.0295 | false positive | 4.17E+08 | 297.1137, 253.1238, 189.0560, 145.0662, 133.0662, 121.0659, 121.0294, 119.0504, 107.0504 | 121.0294 | C_18_H_17_O_4_ | 297.11323 | 0.06 | 10 | 100 |
| 327.1241 |  | 9.29 | 121.0295 | false positive | 4.60E+06 | 327.1236, 161.0608, 147.0455, 137.0611, 122.0375, 121.0297, 93.0347, 83.0140 | 121.0297 | C_19_H_19_O_5_ | 327.1238 | 0.88 | 10 | 100 |
| 277.1446 |  | 9.37 | 165.0193 | false positive | 4.15E+05 | 165.0197, 149.0235, 129.3841, 113.2569 | 165.0197 | C_16_H_21_O_4_ | 277.14453 | 0.33 | 6 | 85.25 |
| **221.0821** |  | **9.49** | **121.0295, 147.0089** | **MiBP^a^ (Level 1)** | **1.74E+06** | **221.0823, 177.0924, 147.0087, 141.0191, 134.0376, 121.0296, 113.0245, 109.0657, 71.0503** | **121.0296, 147.0087** | **C_12_H_13_O_4_** | **221.08193** | **0.72** | **6** | **100** |
| 301.072 |  | 9.68 | 121.0295 | ***isomers of 1,2 benzenedicarboxylic acid, 1-(2-carboxyethyl-cyclohexadiene) methyl ester (level 3)*** | 3.87E+07 | 301.0720, 242.0590, 196.0015, 164.0118, 151.0038, 136.0168, 134.0375, 108.0219, 107.0140, 83.0139 | 121.0294, 165.0194 | C_16_H_13_O_6_ | 301.07176 | 0.76 | 10 | 99.79 |
| **221.082** |  | **9.97** | **121.0295, 147.0089** | **MBP^a^ (Level 1)** | **2.50E+06** | **177.0925, 147.0084, 134.0378, 121.0296, 71.0503, 69.0347** | **121.0296, 147.0084** | **C_12_H_13_O_4_** | **221.08193** | **0.45** | **6** | **99.13** |
| 327.1241 |  | 9.99 | 121.0295 | false positive | 1.62E+06 | 327.1253, 312.1014, 268.1113, 205.0512, 183.0122, 161.0612, 147.0455, 121.0296, 107.0505 | 121.0296 | C_19_H_19_O_5_ | 327.1238 | 0.97 | 10 | 97.84 |
| 301.0721 |  | 10.09 | 165.0193 | false positive | 1.47E+06 | 301.0726, 221.0458, 196.0013, 164.0118, 151.0040, 136.0164, 134.0377, 108.0217, 107.0139, 83.0139, 65.0031 | 165.0185 | C_16_H_13_O_6_ | 301.07176 | 0.97 | 10 | 98.18 |
| **293.14** |  | **10.4** | **121.0295, 147.0089** | **MEHHP^a^ (Level 1)** | **4.01E+06** | **143.1081, 121.0297** | **121.0297, 147.0088** | **C_16_H_21_O_5_** | **293.13945** | **1.73** | **6** | **100** |
| 357.1348 |  | 10.65 | 121.0295 | false positive | 4.16E+06 | 168.0431, 136.0531, 121.0296, 83.0140 | 121.0296 | C_20_H_21_O_6_ | 357.13436 | 1.13 | 10 | 97.51 |
| 293.1399 |  | 11.57 | 121.0295 | false positive | 6.96E+06 | 216.1157, 177.0923, 162.0688, 115.0402, 71.0503 | 121.0294 | C_16_H_21_O_5_ | 293.13945 | 1.42 | 6 | 100 |
| 341.1034 |  | 11.6 | 121.0295 | false positive | 9.62E+06 | 341.1036, 176.0480, 175.0402, 159.0453, 147.0452, 123.0454, 121.0296, 93.0347, 69.0347 | 121.0296 | C_19_H_17_O_6_ | 341.10306 | 0.88 | 11 | 99.73 |
| 291.1242 |  | 11.64 | 121.0295 | false positive | 2.85E+06 | 143.1077, 121.0297, 113.0972, 99.0814, 71.0502, 57.0343 | 121.0297 | C_16_H_19_O_5_ | 291.1238 | 1.41 | 7 | 98.34 |
| 307.1554 | 11.92 | 11.71 | 121.0295, 147.0089, 165.0195 | ***mono-3,4-dimethyl-5-ethyl-6-hydroxyhexyl phthalate (level 2)*** | 5.24E+06 | 121.0296 | 121.0296, 147.0088 | C_17_H_23_O_5_ | 307.1551 | 1.07 | 6 | 98.17 |
| **255.0667** |  | **11.9** | **121.0295, 147.0089** | **MBzP^a^ (Level 1)** | **9.86E+05** | **237.1492, 193.1601, 149.0244, 147.0089, 121.0294, 107.0503, 81.2303** | **121.0294, 147.0089** | **C_15_H_11_O_4_** | **255.06628** | **1.62** | **10** | **86.05** |
| 307.1551 |  | 12.07 | 121.0295, 147.0089 | ***isomers of mono-2-ethyl-6-hydroxyoctyl phthalate (level 3)*** | 2.58E+06 | 121.0296 | 121.0296, 147.0087 | C_17_H_23_O_5_ | 307.1551 | 0.67 | 6 | 100 |
| 321.1711 |  | 12.14 | 121.0295 | false positive | 6.23E+05 | 173.1185, 148.0536, 127.1129, 121.0296, 57.0342 | 121.0296 | C_18_H_25_O_5_ | 321.17075 | 1.23 | 6 | 100 |
| 321.1346 |  | 12.15 | 121.0295 | false positive | 7.70E+05 | 173.1185, 127.1129, 121.0297, 57.0342 | 121.0297 | C_17_H_21_O_6_ | 321.13436 | 0.69 | 7 | 100 |
| 305.1397 |  | 12.33 | 121.0295, 147.0089 | ***isomers of mono-3-propyl-4-oxo-hexyl phthalate (level 3)*** | 3.29E+06 | 163.0405, 145.0297, 121.0296, 119.0505, 117.0346, 113.0975, 71.0503 | 121.0296, 147.0095 | C_17_H_21_O_5_ | 305.13945 | 0.96 | 7 | 98.17 |
| 351.1818 |  | 12.34 | 121.0295, 165.0193 | ***isomers of mono 1-propyl-2,4-dimethyloctyl phthalate (level 3)*** | 6.14E+06 | 336.1576, 321.1347, 277.1450, 260.1422, 167.0351, 152.0480, 151.0402, 137.0244, 96.9601 | 121.0292, 165.0194 | C_19_H_27_O_6_ | 351.18131 | 1.37 | 6 | 97.66 |
| 263.1287 |  | 12.47 | 165.0193 | false positive | 6.01E+05 | 195.0660, 180.0429, 165.0195, 137.0243 | 165.0195 | C_15_H_19_O_4_ | 263.12888 | -0.61 | 6 | 86.04 |
| 305.1399 | 12.57 | 12.54 | 121.0295, 147.0089 | ***mono-3-propyl-4-oxo-hexyl phthalate (level 2)*** | 2.30E+06 | 121.0296 | 121.0296, 147.0094 | C_17_H_21_O_5_ | 305.13945 | 1.36 | 7 | 98.17 |
| 307.1558 |  | 12.56 | 121.0295, 165.0193 | ***isomers of mono-2-ethyl-6-hydroxyoctyl phthalate (level 3)*** | 3.80E+05 | 233.0820, 219.0665, 218.0589, 215.0714, 187.0770, 165.0194, 137.0244, 121.0296 | 121.0296, 165.0194 | C_17_H_23_O_5_ | 307.1551 | 2.16 | 6 | 84.47 |
| 321.1711 |  | 12.58 | 121.0295 | false positive | 1.26E+06 | 173.1181. 121.0297 | 121.0297 | C_18_H_25_O_5_ | 321.17075 | 1.14 | 6 | 83.71 |
| 307.119 |  | 12.65 | 121.0295, 165.0193 | ***isomers of mono-1-hydroxy-2-oxo-5ethylhexyl phthalate (Level 3)*** | 3.72E+07 | 233.0822, 219.0665, 218.0587, 215.0715, 214.0432, 187.0766, 165.0195 | 121.0293, 165.0195 | C_16_H_19_O_6_ | 307.119 | 1 | 7 | 99.79 |
| 263.1291 |  | 12.65 | 165.0193 | false positive | 8.45E+06 | 233.0821, 218.0587, 215.0714, 205.0868, 187.0276, 178.0276, 166.0277, 165.0197, 159.0819 | 165.0197 | C_15_H_19_O_4_ | 263.12888 | 0.9 | 6 | 98.67 |
| 305.1762 |  | 12.67 | 121.0295 | false positive | 4.56E+06 | 305.1761, 261.1865, 162.0688, 149.0610, 136.0531, 121.0298 | 121.0298 | C_18_H_25_O_4_ | 305.17583 | 1.23 | 6 | 100 |
| 317.1761 |  | 12.67 | 121.0295 | false positive | 5.97E+06 | 317.1763, 299.1655, 273.1864, 229.1602, 135.0453, 123.0454, 122.0375 | 121.0297 | C_19_H_25_O_4_ | 317.17583 | 0.8 | 7 | 97.98 |
| 319.1555 |  | 13.07 | 121.0295 | false positive | 5.05E+05 | 303.1980, 188.3721, 160.2013, 121.0295 | 121.0295 | C_18_H_23_O_5_ | 319.1551 | 1.22 | 7 | 100 |
| 335.1868 |  | 13.38 | 121.0295 | false positive | 2.13E+07 | 177.0923, 162.0688, 157.0873 | 121.0296 | C_19_H_27_O_5_ | 335.1864 | 1.29 | 6 | 99.76 |
| 319.1918 |  | 13.42 | 121.0295 | false positive | 7.68E+06 | 319.1918, 275.2023, 163.0767, 150.0687, 149.0609 | 121.0281 | C_19_H_27_O_4_ | 319.19148 | 0.91 | 6 | 100 |
| 377.1974 |  | 13.48 | 121.0295, 165.0193 | ***isomers of mono-3-butyl-9-carboxynonyl phthalate (level 3)*** | 1.65E+07 | 221.0821, 220.0741, 203.0716, 167.0351, 152,0117 | 121.0296, 165.0190 | C_21_H_29_O_6_ | 377.19696 | 1.05 | 7 | 100 |
| 263.1292 |  | 13.69 | 121.0295 | false positive | 6.63E+05 | 237.2341, 145.0626, 134.0379, 121.0293, 113.0973, 111.0814 | 121.0293 | C_15_H_19_O_4_ | 263.12888 | 1.02 | 6 | 100 |
| **277.1448** |  | **14.47** | **121.0295, 147.0091** | **MEHP^a^ (Level 1)** | **2.96E+06** | **147.0091, 134.0375, 127.1130, 121.0296, 93.0345, 75.0240** | **121.0298, 147.0091** | **C_16_H_21_O_4_** | **277.14453** | **0.99** | **6** | **100** |
| 291.1606 | 15.58 | 15.56 | 121.0295, 147.0089 | ***mono-7-methyloctyl phthalate (level 2)*** | 9.39E+05 | 197.1382, 163.5550, 147.0089, 141.1284, 134.0375, 121.0297 | 121.0297, 147.0089 | C_17_H_23_O_4_ | 291.16018 | 1.38 | 6 | 98.33 |
| 349.1659 |  | 15.9 | 165.0193 | false positive | 2.63E+07 | 275.1293, 219.0665, 180.0430, 167.0351, 166.0274, 137.0244 | 165.0195 | C_19_H_25_O_6_ | 349.16566 | 0.66 | 7 | 99.73 |
| 375.1817 |  | 18.63 | 165.0193 | false positive | 1.12E+07 | 360.1610, 301.1438, 269.1181, 233.0822, 219.0663, 218.0586, 204.0428, 201.0561, 167.0352, 151.0398, 137.0245 | 165.0192 | C_21_H_27_O_6_ | 375.18131 | 0.96 | 8 | 99.68 |
| *197.0642* |  | 6.75 | *124.0396, 151.0220* | *MMP (ring-1,2-^13^C_2_, dicarboxyl-^13^C_2_)* | 1.10E+06 | 182.0241, 151.0218, 137.0246, 124.0397, 123.0730 | 124.0397, 151.0218 | C_6_^13^C_4_H_9_O_4_ | 197.0637 | 2.54 | 6 | 0 |
| *225.0955* |  | 9.96 | *124.0396, 151.0220* | *MBP (ring-1,2-^13^C_2_, dicarboxyl-^13^C_2_)* | 5.50E+06 | 151.0224, 137.0477, 124.0395, 123.0364, 95.0414, 77.0307, 71.0503, 69.0346 | 124.0395, 151.0224 | C_8_^13^C_4_H_13_O_4_ | 225.09535 | 0.61 | 6 | 99.13 |
| *297.1532* |  | 10.4 | *124.0396, 151.0220* | MEHHP (ring-1,2-^13^C_2_, dicarboxyl-^13^C_2_) | 1.60E+07 | 143.1079, 124.0397 | 124.0397, 151.0222 | C_12_^13^C_4_H_21_O_5_ | 297.15287 | 1.01 | 6 | 98.14 |
| *311.1324* |  | 10.85 | *124.0396* | MECPP (ring-1,2-^13^C_2_, dicarboxyl-^13^C_2_) | 1.20E+06 | 237.1861, 159.1029, 145.0618, 124.0397, 113.0973 | 124.0397 | C_12_^13^C_4_H_19_O_6_ | 311.13213 | 1.01 | 7 | 0 |
| 295.1374 |  | 11.64 | 124.0396, 151.0220 | MEOHP (ring-1,2-^13^C_2_, dicarboxyl-^13^C_2_) | 2.00E+07 | 143.1079, 124.0397, 113.0974, 99.0817, 71.0503, 57.0344 | 124.0397, 151.0222 | C_12_^13^C_4_H_19_O_5_ | 295.13722 | 0.9 | 7 | 98.15 |
| *259.0797* |  | 11.9 | *124.0396, 151.0220* | *MBzP (ring-1,2-^13^C_2_, dicarboxyl-^13^C_2_)* | 2.50E+06 | 179.0348, 151.0222, 141.1284, 124.0394, 121.0297, 107.0506, 101.0609, 71.0502 | 124.0394, 151.0222 | C_11_^13^C_4_H_11_O_4_ | 259.0797 | 0.39 | 10 | 0 |
| *251.1112* |  | 12.17 | *124.0396, 151.0220* | *MCyHP (ring-1,2-^13^C_2_, dicarboyxl-^13^C_2_)* | 3.10E+07 | 151.0222, 124.0396, 97.0660, 95.0504 | 124.0396, 151.0222 | C_10_^13^C_4_H_15_O_4_ | 251.111 | 0.63 | 7 | 0 |
| 339.1636 |  | 14.05 | 124.0396, 151.0220 | non-spiked | 8.00E+05 | 219.0663, 176.0118, 141.0923, 133.0659, 124.0396, 119.0503, 84.0215 | 124.0396, 151.0222 | C_14_^13^C_4_H_23_O_6_ | 339.16343 | 0.6 | 7 | 81.67 |
| *281.1581* |  | 14.47 | *124.0396, 151.0220* | *MEHP (ring-1,2-^13^C_2_, dicarboxyl-^13^C_2_)* | 6.00E+07 | 151.0222, 137.0475, 127.1130, 124.0396, 123.0364, 95.0414, 77.0308 | 124.0396, 151.0222 | C_12_^13^C_4_H_21_O_4_ | 281.15795 | 0.46 | 6 | 99.85 |
| *295.1737* |  | 15 | *124.0396, 151.0220* | *7-MMOP (ring-1,2-^13^C_2_, dicarboxyl-^13^C_2_)* | 6.00E+07 | 151.0223, 142.1319, 141.1286, 140.1162, 139.1129, 137.0473, 124.0395, 69.0347 | 124.0395, 151.0223 | C_13_^13^C_4_H_23_O_4_ | 295.1736 | 0.35 | 6 | 0 |
| *281.1581* |  | 15.13 | *124.0396, 151.0220* | *(MOP) (ring-1,2-^13^C_2_, dicarboxyl-^13^C_2_)* | 6.00E+07 | 151.0227, 137.0475, 128.1163, 127.1130, 126.1007, 125.0973, 124.0397, 95.0412, 77.0309 | 124.0397, 151.0227 | C_12_^13^C_4_H_21_O_4_ | 281.15795 | 0.57 | 6 | 0 |
| *309.1894* |  | 17.11 | *124.0396, 151.0220* | *3,7-diMMOP (ring-1,2-^13^C_2_, dicarboxyl-^13^C_2_)* | 1.60E+08 | 156.1475, 155.1443, 154.1319, 153.1289, 151.0221, 139.1129, 137.0474, 124.0398, 69.0345 | 124.0398, 151.0221 | C_14_^13^C_4_H_25_O_4_ | 309.18925 | 0.55 | 6 | 0 |

Peak filter was set up as relative height of no less than 10 % of largest peak

*Italicized names and red color*: isotopically labelled compound

^a^: Confirmed with authentic standards (highlighted with orange)

^b^:Not confirmed with standards (highlighted with green)

Highlighted in yellow color are non-spiked labelled compounds

Table S7. Phthalate metabolites assigned by Compound Discoverer.

| Mass | RT (min) | Formula | Diagnostic ions | Identification Confidence Level | Compound assigned by Compound Discoverer | Compound identification based on predicted fragmentation and retention time |
| --- | --- | --- | --- | --- | --- | --- |
| 236.06859 | 7.758 | C_12_H_12_O_5_ | None | False positive | Mono-(3-oxobutyl)-phthalate | N/A |
| 236.06859 | 7.164 |  | None | False positive |  |  |
| 252.10013  252.10013  252.10013 | 6.053 | C_13_H_16_O_5_ | None | False positive | Mono-(4-hydroxypentyl)-phthalate | N/A |
|  | 12.958 |  | None | False positive |  |  |
|  | 12.411 |  | None | False positive |  |  |
| 280.09487 | 5.72 | C_14_H_16_O_6_ | None | False positive | Mono-(5-carboxypentyl)-phthalate | N/A |
| 280.09496 | 7.786 |  | No MS2 data available | False positive |  |  |
| 280.09493 | 7.349 |  | None | False positive |  |  |
| 266.1152 | 12.807 | C_14_H_18_O_5_ | None | False positive | Mono-(5-hydroxyhexyl)-phthalate | N/A |
| 266.11517 | 6.249 |  | No MS2 data available | False positive |  |  |
| 266.11572 | 5.105 |  | No MS2 data available | False positive |  |  |
| 266.11574 | 8.967 |  | No MS2 data available | False positive |  |  |
| 266.11528 | 13.175 |  | None | False positive |  |  |
| 266.11569 | 6.74 |  | None | False positive |  |  |
| 266.11571 | 12.369 |  | None | False positive |  |  |
| 266.11529 | 12.594 |  | None | False positive |  |  |
| 266.11569 | 8.541 |  | None | False positive |  |  |
| 264.09996 | 6.034 | C_14_H_16_O_5_ | No MS2 data available | False positive | Mono-(5-oxohexyl)-phthalate | N/A |
| 264.09999 | 5.825 |  | No MS2 data available | False positive |  |  |
| 264.09993 | 6.314 |  | None | False positive |  |  |
| 294.11077 | 8.517 | C_15_H_18_O_6_ | None | False positive | Mono-(6-carboxyhexyl)-phthalate | N/A |
| 294.11074 | 6.378 |  | 121.0293 | False positive |  |  |
| 294.11072 | 7.712 |  | None | False positive |  |  |
| 280.13138 | 9.89 | C_15_H_20_O_5_ | No MS2 data available | False positive | (Mono-(6-hydroxyheptyl)-phthalate) | N/A |
| 280.13133 | 12.418 |  | No MS2 data available | False positive |  |  |
| 280.1313 | 7.415 |  | None | False positive |  |  |
| 280.13145 | 12.975 |  | No MS2 data available | False positive |  |  |
| 280.13122 | 6.449 |  | No MS2 data available | False positive |  |  |
| 280.13125 | 6.046 |  | No MS2 data available | False positive |  |  |
| 308.16281 | 11.96 | C_17_H_24_O_5_ | 121.0296,147.0091,165.0195 | 2 | 2-{[(7-Hydroxy-4-methyloctyl)oxy]carbonyl}benzoic acid (mono-hydroxy-isononyl phthalate) | Mono-3,4-dimethyl-5-ethyl-6-oxohexyl |
| 308.16269 | 10.703 |  | 121.0296 | False positive |  | N/A |
| 308.16269 | 7.286 |  | 121.0297 | False positive |  | N/A |
| 308.16292 | 12.967 |  | 121.0293, 165.0195 | 3 |  | Isomers of mono-hydroxy nonyl |
| 308.16286 | 12.37 |  | 121.0296 | False positive |  | N/A |
| 312.19421 | 11.235 | C_17_H_28_O_5_ | None | False positive | mono-4-methyl-7-oxooctyl ester | N/A |
| 298.21469 | 12.414 | C_17_H_30_O_4_ | None | False positive | mono-4-methyloctyl ester | N/A |
| 298.21474 | 7.08 |  | None | False positive |  |  |
| 298.21467 | 12.714 |  | None | False positive |  |  |
| 314.20982 | 12.68 | C_17_H_30_O_5_ | No MS2 data available | False positive | mono-7-hydroxy-4-methyloctyl ester | N/A |
| 314.2097 | 6.688 |  | 121.0299 | False positive |  |  |
| 314.20985 | 7.371 |  | 121.0297 | False positive |  |  |
| 314.20985 | 12.397 |  | 121.0293 | False positive |  |  |
| 314.20985 | 13.091 |  | 121.0293 | False positive |  |  |
| 314.20988 | 12.919 |  | 121.0294 | False positive |  |  |
| 320.16267 | 13.095 | C_18_H_24_O_5_ | 121.0296 | False positive | mono-(2-propyl-6-oxoheptyl ) phthalate | N/A |
| 320.16276 | 11.96 |  | No MS2 data available | False positive |  |  |
| 320.16268 | 12.615 |  | None | False positive |  |  |
| 320.16266 | 13.317 |  | 121.0296 | False positive |  |  |
| 320.16267 | 13.521 |  | 121.0296 | False positive |  |  |
| 322.17823 | 12.888 | C_18_H_26_O_5_ | 121.0296 | False positive | mono-(2-propyl-6-hydroxyheptyl) phthalate | N/A |
| 322.1782 | 8.283 |  | None | False positive |  |  |
| 322.17824 | 12.418 |  | 121.0295 | False positive |  |  |
| 322.17818 | 12.711 |  | 121.0297 | False positive |  |  |
| 322.17824 | 8.197 |  | None | False positive |  |  |
| 306.18351 | 16.094 | C_18_H_26_O_4_ | None | False positive | mono(3,7-dimethyloctyl) phthalate | N/A |
| 306.1835 | 10.618 |  | None | False positive |  |  |
| 306.18351 | 13.187 |  | 121.0297 | False positive |  |  |
| 306.18346 | 11.89 |  | None | False positive |  |  |
| 306.18351 | 12.95 |  | 121.0295 | False positive |  |  |
| 306.18342 | 12.415 |  | 121.0295 | False positive |  |  |
| 252.06355 | 5.557 | C_12_H_12_O_6_ | No MS2 data available | False positive | Mono(3-carboxypropyl) phthalate | N/A |
| 252.0637 | 6.786 |  | None | False positive |  |  |
| 252.06365 | 6.3 |  | None | False positive |  |  |
| 306.14702 | 12.588 | C_17_H_22_O_5_ | 121.0296,147.0087 | 2 | mono-(4-methyl-7-oxooctyl) phthalate | Mono-3-propyl-4-oxohexyl |
| 306.1471 | 11.662 |  | None | False positive |  | N/A |
| 306.14703 | 12.816 |  | 121.0298 | False positive |  | N/A |
| 306.14718 | 10.399 |  | None | False positive |  | N/A |
| 306.14697 | 6.623 |  | None | False positive |  | N/A |
| 306.14702 | 12.405 |  | 121.0296 | False positive |  | N/A |
| 322.14176 | 6.735 | C_17_H_22_O_6_ | 121.0297 | False positive | mono-1-methyl-7-carboxyheptyl phthalate | N/A |
| 322.14201 | 11.974 |  | None | False positive |  |  |
| 322.142 | 12.425 |  | 121.0297 | False positive |  |  |
| 308.12627 | 12.903 | C_16_H_20_O_6_ | 121.0296,165.0196 | 3 | mono-2-ethyl-5-carboxypentyl phthalate | Isomers of mono-2-ethyl-5-carboxypentyl |
| 308.12636 | 10.967 |  | 121.0296,134.0382 | 3 |  | Isomers of mono-2-ethyl-5-carboxypentyl |
| 308.12623 | 6.416 |  | None | False positive |  | N/A |
| 308.12647 | 11.927 |  | None | False positive |  | N/A |
| 294.14717 | 10.502 | C_16_H_22_O_5_ | None | False positive | mono-2-ethyl-5-hydroxyhexyl phthalate | N/A |
| 294.14717 | 11.773 |  | None | False positive |  |  |
| 292.13149 | 9.712 | C_16_H_20_O_5_ | None | False positive | mono-2-ethyl-5-oxohexyl phthalate | N/A |
| 292.13156 | 11.865 |  | None | False positive |  |  |
| 292.13136 | 12.81 |  | None | False positive |  |  |
| 238.08433 | 12.414 | C_12_H_14_O_5_ | No MS2 data available | False positive | mono-3-hydroxybutyl phthalate | N/A |
| 292.16784 | 15.967 | C_17_H_24_O_4_ | 121.0296 | False positive | mono-7-methyloctyl phthalate | N/A |
| 292.16786 | 11.522 |  | None | False positive |  |  |
| 292.1678 | 10.958 |  | No MS2 data available | False positive |  |  |
| 292.1678 | 16.216 |  | No MS2 data available | False positive |  |  |
| 292.1678 | 12.277 |  | 121.0296 | False positive |  |  |
| 292.16783 | 15.749 |  | 121.0296,134.0373,147.0089 | 2 |  | Mono-7-methyloctyl |
| 292.16783 | 12.414 |  | 121.0298 | False positive |  | N/A |
| 292.16783 | 8.52 |  | No MS2 data available | False positive |  | N/A |
| 256.07385 | 6.943 | C_15_H_12_O_4_ | 121.0296 | False positive | monobenzyl phthalate |  |
| 256.07377 | 5.674 |  | None | False positive |  |  |
| 256.07399 | 12.16 |  | 121.0296,147.0087 | 1 |  |  |
| 256.07397 | 13.159 |  | None | False positive |  |  |
| 248.10515 | 7.322 | C_14_H_16_O_4_ | None | False positive | monocyclohexyl phthalate | N/A |
| 194.05791 | 5.55 | C_10_H_10_O_4_ | 121.0296,134.0373 | 2 | Monoethyl phthalate | Monoethyl |
| 194.05789 | 5.847 |  | 121.0296,134.0373 | 2 |  | Monoethyl |
| 194.05774 | 6.902 |  | 121.0296,134.0373 | 2 |  | Monoethyl |
| 194.05793 | 7.109 |  | 121.0296,134.0373 | 2 |  | Monoethyl |
| 194.0579 | 6.632 |  | 121.0296,134.0373,147.0087 | 1 |  | Monoethyl |
| 194.05787 | 6.076 |  | 121.0296,134.0373 | 2 |  | Monoethyl |
| 194.05794 | 4.207 |  | None | False positive |  | Monoethyl |
| 194.05791 | 4.98 |  | 121.0296,134.0373 | 2 |  | Monoethyl |
| 264.13647 | 7.856 | C_15_H_20_O_4_ | None | False positive | monoheptyl phthalate | N/A |
| 264.13661 | 13.786 |  | None | False positive |  |  |
| 264.1365 | 12.902 |  | 165.0192 | False positive |  |  |
| 264.13647 | 8.867 |  | None | False positive |  |  |
| 264.13652 | 12.455 |  | No MS2 data available | False positive |  |  |
| 264.13643 | 6.973 |  | None | False positive |  |  |
| 264.13648 | 6.807 |  | None | False positive |  |  |
| 264.13646 | 6.443 |  | None | False positive |  | N/A |
| 264.13661 | 13.913 |  | 121.0298,134.0379 | 1 |  | Monoheptyl |
| 250.12082 | 13.249 | C_14_H_18_O_4_ | None | False positive | Monohexyl Phthalate | N/A |
| 250.12082 | 13.379 |  | None | False positive |  |  |
| 250.12077 | 7.05 |  | None | False positive |  |  |
| 250.12078 | 7.759 |  | None | False positive |  |  |
| 180.04234 | 5.017 | C_9_H_8_O_4_ | 121.0296,134.0379 | 3 | Monomethyl phthalate |  |
| 180.04235 | 4.281 |  | None | False positive |  | N/A |
| 180.04231 | 4.385 |  | 134.0374 | False positive |  | N/A |
| 222.0894 | 6.75 | C_12_H_14_O_4_ | None | False positive | mono-n-butyl phthalate | N/A |
| 222.0894 | 10.078 |  | 121.0296,134.0375,147.0083 | 1 |  | Monobutyl |
| 222.0894 | 9.613 |  | 121.0296,134.0376 | 1 |  | Monoisobutyl |
| 222.08934 | 6.202 |  | None | False positive |  | N/A |
| 278.15206 | 9.469 | C_16_H_22_O_4_ | None | False positive | Mono-2-ethylhexyl phthalate | N/A |
| 278.15197 | 10.799 |  | None | False positive |  | N/A |
| 278.15226 | 14.689 |  | 121.0296,134.0373,147.0089 | 1 |  | Mono-2-ethylhexyl |
| 236.10513 | 7.411 | C_13_H_16_O_4_ | None | False positive | Mono-n-pentyl phthalate | N/A |
| 208.07357 | 5.338 | C_11_H_12_O_4_ | None | False positive | Monopropyl phthalate | N/A |
| 208.07358 | 5.705 |  | None | False positive |  |  |
| 208.07356 | 6.036 |  | None | False positive |  |  |
| 208.07358 | 5.046 |  | None | False positive |  |  |
| 208.07346 | 4.385 |  | None | False positive |  |  |
